# Emotion Recognition Deficits in Children and Adolescents with Psychopathic Traits: A Systematic Review

**DOI:** 10.1007/s10567-023-00466-z

**Published:** 2024-01-19

**Authors:** Beatriz Díaz-Vázquez, Laura López-Romero, Estrella Romero

**Affiliations:** Department of Clinical Psychology and Psychobiology, Facultade de Psicoloxía, Universidade de Santiago de Compostela, Campus Vida, Santiago de Compostela, Spain

**Keywords:** Emotion recognition, Psychopathic traits, Childhood, Adolescence, Attention bias

## Abstract

Children and adolescents with psychopathic traits show deficits in emotion recognition, but there is no consensus as to the extent of their generalizability or about the variables that may be moderating the process. The present Systematic Review brings together the existing scientific corpus on the subject and attempts to answer these questions through an exhaustive review of the existing literature according to PRISMA 2020 statement. Results confirmed the existence of pervasive deficits in emotion recognition and, more specifically, on distress emotions (e.g., fear), a deficit that transcends all modalities of emotion presentation and all emotional stimuli used. Moreover, they supported the key role of attention to relevant areas that provide emotional cues (e.g., eye-region) and point out differences according to the presence of disruptive behavior and based on the psychopathy dimension examined. This evidence could advance the current knowledge on developmental models of psychopathic traits. Yet, homogenization of the conditions of research in this area should be prioritized to be able to draw more robust and generalizable conclusions.

**Supplementary Information:**

The online version contains supplementary material available at 10.1007/s10567-023-00466-z.

## Introduction

Psychopathic personality, defined as a constellation of interpersonal (e.g., superficial charm, grandiosity), affective (lack of remorse, callousness) behavioral/lifestyle (e.g., irresponsibility, impulsivity) and arguably antisocial traits (e.g., poor behavioral control, early behavioral problems) (Hare & Neumann, 2008), constitute one the best predictors of severe, chronic and difficult-to-treat antisocial behavior, with an important economic and social burden (Reidy et al., 2015). In an effort to gain deeper insights into the emergence of the most serious, aggressive and persistent pattern of child and youth conduct problems (CP), the study of psychopathic personality has been downward extended to early developmental stages. This line of research has allowed to collect extensive evidence on usefulness and viability of psychopathic traits across early childhood (i.e., preschool years; e.g., Waller & Hyde, 2018), middle childhood (i.e., elementary school years; e.g., Gorin et al., 2019) and adolescence (e.g., Lynam et al., 2009). Previous research conducted in young samples was mainly focused on the affective dimensions of psychopathic traits (i.e., CU traits), largely examined as a putative precursor of adult psychopathy (Hyde & Dotterer, 2022). In this regard, an extensive line of research has supported CU traits as a potential identifier of an etiological and clinically distinctive subgroup of problematic children^1^ (see Frick et al., 2014). As a result, a new specifier, largely based on the CU conceptualization, has been added for CD in the latest edition of the Diagnostic and Statistical Manual of Mental Disorders (DSM-5; American Psychiatric Association—APA, 2013; i.e., “with limited prosocial emotions”), and the International Classification of Diseases (ICD-11; World Health Organization—WHO, 2019; i.e., “limited vs. prosocial emotions”).

Regardless the advances achieved from the CU conceptualization, the study of psychopathic personality in childhood and adolescence has been enriched from a multidimensional perspective, involving a constellation of interpersonal [grandiose-manipulative (GM)], affective (CU) and behavioral traits [impulsive-need of stimulation (INS)]. So far, compelling evidence on early identification, relative stability and predictive value of the psychopathic constellation has been consistently provided (Salekin et al., 2018). Beyond old and current debates about which dimension(s) should be considered in developmental models of CP (see Frick, 2022; Salekin, 2022), additional support for considering interpersonal (GM) and behavioral traits (INS)^2^ when defining and studying psychopathic personality has been increasingly collected (see Salekin, 2017, 2022). Hence, some recent studies have reinforced psychopathic traits, conceptualized as a multidimensional construct, as a relevant predictor in the development of serious CP and other forms of child and adolescent maladjustment (e.g., Bergstrøm & Farrington, 2022; Burke et al., 2022; Colins et al., 2022; Fanti et al., 2018; López-Romero et al., 2021, 2022). A critical question in the field is, therefore, whether including other dimensions of psychopathy may increase our knowledge about the construct, particularly clarifying how all affective, interpersonal, and behavioral dimensions develop, and which etiological processes might be underlying. If psychopathic personality indeed identifies a subgroup of children and adolescents with more serious and persistent CP, disentangling its potential distinctive etiological pathways will enrich developmental models of both psychopathic traits and disruptive behavior.

### Unraveling the Developmental Basis of Psychopathic Traits: The Role of Emotion Recognition

Probably due to the prominence of the affective traits in the conceptualization and manifestation of psychopathic personality, affective impairments have been largely researched in relation to psychopathic traits at the neurobiological, cognitive, emotional and behavioral levels (Blair, 2013). Developmental models of psychopathy have commonly pointed to an amygdala dysfunction (e.g., Blair, 2003a), which is involved in emotion recognition (Phelps & LeDoux, 2005), a process that also seems to be affected in individuals high on psychopathic traits (Dawel et al., 2012). Emotion recognition refers to the ability to attribute emotional states in others, based on the identification of emotional cues relevant for socialization. Accurately processing emotional expressions, particularly facial expressions, is critical for everyday functioning as it facilitates appropriate interpersonal communication (Marsh & Blair, 2008), promotes shared affective experience (Hinnant & O’Brien, 2007), and motivates prosocial behavior (Marshall & Marshall, 2011). Conversely, deficits in emotion recognition may lead to dysfunctional interpersonal relationships and social adjustment (Kyranides et al., 2020), particularly when deficits in recognition affect distress emotions (e.g., fear, sadness). Hence, failing to recognize others’ distress may hinder the development of empathic concern through a process that would imply the absence of discomfort that typically follows wrong behaviors (e.g., guilt). This, in turn, would restrain the inhibition of those behaviors that may cause distress in others (e.g., Kochanska et al., 2010). Accordingly, deficits in emotion recognition have been suggested as a potential link between psychopathic traits and different forms of behavioral maladjustment (e.g., CP, antisocial behavior) commonly observed in high psychopathic individuals (Frick et al., 2014; Salekin, 2017).

Whether emotion recognition deficits are specific to distress emotions or reflect a more pervasive emotional impairment has been a question over debate. From one perspective, Blair (1995, 2006) suggested that psychopathy would be marked by *specific* deficits in the recognition of both fear and sadness. This would result in the no-experience of aversion after a behavior that may cause distress in others, allowing individuals with psychopathic traits to behave in a self-gratifying and goal-directed manner, without the negative consequence of feeling guilty and bad. Overall, these deficits would partially explain the callous, unremorseful and deceitful behavior in psychopathic personality. From an alternative approach, Dadds et al. (e.g., Dadds et al., 2006, 2008) have proposed a dysfunction on attentional mechanisms that would underlie emotion recognition deficits. More specifically, attentional deficits to socially relevant cues (i.e., the eyes, with some new evidence also for the mouth; Demetriou & Fanti, 2022), would serve as the basis for the emotion recognition deficits observed in high psychopathic individuals (Dadds et al., 2011, 2014). From this perspective, impairments in emotion recognition would be more *pervasive* rather than specific and would derive a more generalized deficit in socio-emotional functioning (Dawel et al., 2012).

Both theoretical approaches found support in previous meta-analytic studies, which mainly examined results from adult populations. The first documented meta-analysis on the topic provided support for a specific deficit in recognizing fear and, to a lesser extent, sadness from facial expressions in antisocial individuals, with no moderation of psychopathic traits (Marsh & Blair, 2008). Later studies reported a more pervasive deficit, with impairments in the recognition of multiple emotions (Wilson et al., 2011), also when more than facial cues (i.e., vocal, postural) were examined (Dawel et al., 2012). From these meta-analytic studies, only Dawel et al. (2012) distinguished between adult and young samples—including both middle-school children and adolescents, who showed deficits for all emotions, and particularly anger, fear and sadness, with greater effects for fear. Yet, most analyses were conducted for the total sample, with most adults from the forensic setting, and most children/adolescents from community or clinical settings. No additional distinction was made between child and adolescent samples, and no other relevant moderators (e.g., gender, age, sample type, the presence -or not- of concurrent disruptive behavior) were examined.

### Emotion Recognition and Psychopathic Traits in Childhood and Adolescence

The multiple studies published so far in child and adolescent samples have yielded, to date, mixed results, probably due to a variety of designs, sampling procedures and methods that makes it difficult to extract firm conclusions (Northam & Dadds, 2020). Mirroring results from adult samples (e.g., Brislin & Patrick, 2019; Demetrioff et al., 2017; Kyranides et al., 2020), some studies provided additional evidence for the specific deficit in the recognition of fear (e.g., Fairchild et al., 2009), and other distress emotions, including anger or sadness (e.g., Muñoz, 2009; Powell et al., 2023). Others, in contrast, have found more pervasive deficits across different emotions including disgust or happiness (e.g., Kahn et al., 2017). There is also opposite evidence, with some studies showing increased recognition for distress emotions (e.g., Schwenck et al., 2014), or even no association between psychopathic traits and emotion recognition (e.g., Martin-Key et al., 2020). Understanding the diversity of results, and their potential causes and explanations, is of uttermost importance to clarify how psychopathic traits develop, and which mechanisms may boost or restrain their negative consequences. In this regard, applied implications from this knowledge may differ depending on whether emotion recognition deficits indeed exist, whether they are specific to one or two emotions (e.g., fear or sadness), or whether they reflect a more pervasive impairment in socioemotional functioning and the interpretation of others emotional states. Related with this last hypothesis, studies examining attention deficits related to emotion recognition also yielded mixed results, particularly when using eye-tracker methodologies (Demetriou & Fanti, 2022). Thus, even though there was evidence of reduced attention to other’s eyes in children and adolescents high on CU traits (e.g., Dadds et al., 2006, 2008; Martin-Key et al., 2018), some studies also revealed no association between CU traits and attention to the eyes (e.g., Bedford et al., 2017; Muñoz et al., 2021). In addition, the inclusion of other relevant face areas, such as the mouth, has made these results even more complex, with some studies suggesting that the deficits are more specific to the eye-region (e.g., Demetriou & Fanti, 2022) whilst others suggest that emotions linked to eye-region deficits (i.e., fear and anger) could differ from those observed in the mouth-region (i.e., sadness) (Hartmann & Schwenck, 2020).

### The Present Study

The pattern of mixed results obtained in previous research raises the need to systematically organize the current knowledge, providing compelling evidence about well-established findings, and identifying the gaps and inconsistencies that should be addressed in future research. This systematic review is devoted to accurately examining the association between psychopathic traits, addressing all its dimensions, and emotion recognition deficits in young samples, including early childhood, middle-childhood, and adolescence (mean age up to 18 years old). As it might be expected, CU traits have derived most of the research aimed at understanding the aforementioned impairments in emotion recognition and processing (see Northam & Dadds, 2020). However, as evidence has been collected on the multidimensionality of the construct (Salekin, 2017, 2022) studies addressing other psychopathy dimensions would be also considered. This may shed new light on the nature of emotion recognition deficits in psychopathic personality, leading to disentangle some of its mechanistic trends. It would also help to clarify whether previous findings on CU traits can be extrapolated to other psychopathy dimensions, whether there might be specific deficits for specific dimensions or whether a combination of high interpersonal, affective, and behavioral psychopathic traits may identify a distinctive etiological subgroup.

All studies published to date have been taken into account, with no time restrictions imposed. Even though there are some previous meta-analyses on this topic, they have been published more than 10 years ago, two of them considered together young and adult samples (Marsh & Blair, 2008; Wilson et al., 2011), and one distinguished between mid-child/adolescents and adult samples (Dawel et al., 2012). However, the study from Dawel et al. (2012) did not cover any study in preschool samples, did not distinguish between children and adolescents, and did not account for any other potential variables relevant to understand the association between emotion recognition and psychopathic traits in youngsters, such as the sample type or the co-occurrence of CP (Dawel et al., 2012). Therefore, all studies published up to 2022 were examined, and several methodological variables and sample characteristics of studies were compared in an attempt to account for the discrepancies observed in previous research. Due to the importance that attention deficits may have in the emotion recognition impairments observed in individuals high on psychopathic traits (Dadds et al., 2006), and with the aim to provide additional clarification in the aforementioned theoretical approaches, studies examining attention biases in the context of emotion recognition and psychopathic personality were also included.

More specifically, the present systematic review aims to respond to the following questions:

1. Are there emotion recognition deficits in children and adolescents with different levels of psychopathic traits?
2. Are emotion recognition deficits related to specific emotions (e.g., fear, sadness) or do they respond to a more pervasive emotional impairment?
3. Are there specific deficits associated to different psychopathy dimensions?
4. Are emotion recognition deficits specifically related with attention biases to relevant areas showing emotional and/or distress cues? (e.g., eye region, mouth region)
5. Are there specific differences according to age (i.e., childhood versus adolescence), gender (i.e., boys, girls), sample type (i.e., community-based, forensic, clinical-referred) and the co-occurrence of different forms of disruptive behavior including CP, conduct disorder (CD) and oppositional defiant disorder (ODD)?

In accordance with previous literature, we anticipate (a) an association between emotion recognition and psychopathic traits; (b) the presence of more pervasive rather than specific deficits; (c) the influence of the different dimensions of psychopathy, (d) a relevant role of attention biases in emotion recognition deficits; and (e) the moderating role of socio-demographic variables, including sex and age, as well as the existence of clinical diagnoses (CD, ODD) or subclinical symptoms (CP).

In sum, this review is aimed to organize the available evidence in this field, by providing a comprehensive guide of studies and by addressing multiple sources of variability. Ultimately, this review is expected to identify shared conclusions across studies, and to delineate some guidelines to homogenize future protocols and favor additional replication.

## Method

### Systematic Search Strategy

The present review was performed according to the actualized Preferred Reporting Items for Systematic Reviews and Meta-Analyses (PRISMA) guidelines (Page et al., 2021). The *PRISMA* Checklist is provided in Table S1, available online. A protocol for this study was developed and registered on *PROSPERO* (Registration number: CRD42021276769).

A search strategy (available on Prospero and Supplement X) was developed to cover the key elements of this systematic review, including (1) psychopathic traits (callous* unemotion* or CU or psychopathy or psychopathic), (2) emotion recognition (emotion* recognition or emotion* process* or emotion* identification or eye gaze or eye track* or eye fix* or facial emotion* or emotion* attent*), and (3) the developmental period (child* or adolesc*). Note that emotion processing was also included in the search strategy since some studies used indistinctively emotion recognition and processing to examine specific deficits in the recognition of basic emotions. The search to identify relevant literature was conducted on September 22nd, 2021 on PsycInfo, Scopus, PubMed and Web of Science (WOS) databases. An update of the systematic search was conducted on December 23, 2022 in order to include all published studies up to 2022. The same search terms were applied across all databases with adjustments made to accommodate the specific requirements of the search sites (see Table 1). We did not impose a start data as most studies in this area have been published in the past 20 years, and it was our intention to provide a complete picture of the state of the art. Publications were restricted to peer review journal articles to ensure a minimum threshold for quality. In addition to the electronic search, the reference lists of the included studies were also checked to identify other potentially eligible studies.

**Table 1:** Systematic search strategy

Database	PsycInfo	Scopus	Pubmed	WOS
Keywords	L1. (callous* unemotion* or CU or psychopathy or psychopathic) #^a^ L2. (emotion* recognition or emotion* process* or emotion* identification or eye gaze or eye track* or eye fix* or facial emotion* or emotion* attent*) # L3. (child* or adolesc*)			

Fields	Any field (ALL)	TITLE-ABS-KEY	All fields	All fields
Results^b^	939	321	1044	1273
Filters	1. Language: English, Spanish 2. Age: 0–18 3. Peer Review 4. Humans (male, female, inpatient, outpatient)	1. Language: English	1. Language: English, Spanish 2. Humans 3. Age: 0–18	1. Language: English, Spanish

^a^#= AND

^b^Results based on the updated systematic search, conducted on December 23, 2022

### Eligibility Criteria

This review aimed to include all published studies, in English or Spanish, that examined the relationship between psychopathic traits and emotion recognition in children and adolescents (mean age up to 18). Studies including participants aged ≥ 18 were included as long as they were based on a well-established adolescent sample (e.g., high-school students; forensic juvenile samples that in some contexts may involve participants up to age 21). The review included cross-sectional and longitudinal studies, with both correlational and/or between-groups designs. Specific inclusion criteria for studies in this review were:

1. Studies should include a validated measure of psychopathic traits, as well as a valid recognition measure (lab task), with normed or validated stimuli (i.e., facial, vocal, postural expression) for at least one or more basic emotions (e.g., anger, disgust, fear, happiness, sadness and surprise; Ekman, 1992).
2. Samples might be community, at risk, forensic or clinical, or any combination thereof. Forensic samples are defined as populations that have been in contact with the juvenile justice system; clinical samples include participants that have been diagnosed with some mental disorder, particularly CD or ODD; at risk samples include participants from disadvantaged environments, as well as those with high levels of CP that have not yet been in contact with juvenile justice systems not either have received a mental disorder diagnosis, and community samples include participants who do not meet criteria for forensic, clinical or at risk samples.
3. Studies defining psychopathic traits as a dichotomous variable (e.g., high CU traits), should also include a control group. The control group may be a community or a matched forensic, at risk or clinical sample with distinctive levels of psychopathic traits (e.g., low CU traits).
4. Studies must be full-text papers published in peer-reviewed journals in both Spanish and English.

Studies were excluded if they were based on adult samples (mean age < 18), or whether they explicitly included participants with known cognitive impairments that are likely to influence emotion recognition, including brain injuries, neurodevelopmental disorders (e.g., autism spectrum disorder; ASD) or current substance abuse. Studies including between-groups designs with participants meeting criteria for one or more of the aforementioned impairments were retained as long as it was possible to extract specific data for participants with psychopathic traits who do not meet criteria for those impairments. Studies exclusively based on populations with an attention deficit hyperactivity disorder (ADHD) diagnosis were also excluded. Yet, considering the high co-occurrence rates between CP and ADHD (Hudec & Mikami, 2017), studies were retained if they were based on populations with CP, who may also meet criteria for CD or ODD, even though they reported the inclusion of participants with comorbid ADHD (*k* = 17). To gain clarity about the potential influence of ADHD symptoms in emotion recognition deficits related to psychopathic traits, the percentage of participants with ADHD will be extracted, and information about control analysis (or the absence of) will be explicitly provided.

### Study Identification and Selection

Title and abstract were screened by two researchers, and excluded if they did not meet the inclusion criteria. Both researchers were blinded to each other’s decisions and the first study selection was put in common once the study selection phase was finished. RefWorks was independently used by both researchers as a reference manager tool. Of the 3215 abstracts screened, 74 were included for full-text review, which was also performed by two researchers who worked independently. If studies met the inclusion criteria, they were finally included in the review. If they violated some of the eligibility assumptions, they were classified based on the reason of exclusion (e.g., adult samples, emotion processing instead of emotion recognition, no measures of psychopathic traits, no lab task for emotion recognition, participants with neurodevelopmental disabilities). At the full-text level, articles were excluded if they: Included participants with a mean age that exceed the 18 years of age (*k* = 3), measured emotion recognition through questionnaires or scenarios with additional contextual cues that require emotional inferences (*k* = 3), examined complex instead of basic emotions (*k* = 1), emotion recognition was computed as a function of a global measure (e.g., moral reasoning, emotional understanding) (*k* = 5), or it was restricted to inform about owns’ emotional state in response to emotional content (e.g., emotional responsiveness) (*k* = 2), emotion recognition deficits were not (exclusively) linked to psychopathic traits (*k* = 4), assessed emotional processing instead of emotion recognition (*k* = 4), and there was evidence of substance abuse (*k* = 2). If the studies met the inclusion criteria but sufficient data was not available to extract the main results, data were directly requested from the authors by email.

At all stages of the review process, disagreements were solved by discussion and consensus and, when needed, a third researcher settled the unresolved disagreements. The inter-rater agreement Cohen’s kappa was used to compare agreement between the researchers regarding the decision to include or exclude the eligible studies, and interpreted as ≤ 0, no agreement, 0.01–0.20, none to slight, 0.21–0.40 fair, 0.41–0.60 moderate, 0.61–0.80 substantial, 0.81—1.00 almost perfect agreement (McHugh, 2012).

### Quality Assessment

Two independent reviewers assessed the quality of included studies using the Appraisal Tool for Cross-Sectional Studies (AXIS) (Downes et al., 2016), which contains 20 questions regarding introduction, methods, results and discussion of each study. Each question could be answered with “*yes*” (1 point) or “*no*”/“*don’t know*” (0 points). Longitudinal studies were assessed via the Critical Appraisal Skills Program (CASP), a 12-question checklist that addresses different aspects concerning the objectives, sample recruitment, measurement, attrition, results and implications. Disagreements on studies’ quality assessment were solved by consensus.

### Data Extraction and Synthesis

Relevant data for each included article were added to an extraction sheet developed for this review and refined when necessary. Data extraction included participants characteristics (N [females], sample type and age range [mean age]), psychopathic and/or CU traits measure and informant, emotion recognition task specificities (i.e., type of stimuli, emotions assessed and measurement outcome), and main results specifically related with the objectives of this review. For correlational studies we extracted results focused on the relationship between psychopathic traits and emotion recognition, whilst in between-group designs we focused on results obtained in comparisons between the high psychopathic and the comparison groups. Between-group designs where psychopathic traits were examined as potential covariates were also considered as long as it was possible to extract specific data on the effect of psychopathic traits on emotion recognition. For studies assessing attention biases, information about the Areas of interest (AOI) and the measurement outcome was also extracted. Studies were organized by their focus on specific CU traits (*k* = 26) (Table 2) or multidimensional psychopathic traits (Table 3), and the inclusion of attention biases measurement (Table 4). Additional information about main study characteristics was also collected, including: the main purpose of the study, the study design, sample definition, percentage (%) of males, location and ethnicity, psychopathy dimension, emotion recognition task specificities (i.e., stimuli, exposure duration, number of blocks and trials, and response format), and the inclusion (or not) of attention biases analyses including, when available, the type of measure and/or the measurement device (Table S3).

**Table 2:** Studies examining emotion recognition in relation to CU traits

Study	Participants			CU measure (informant)	Emotion recognition			Main outcome specific to emotion recognition
	*N* (female)	Sample type	Age range (*M*)		Type of stimuli	Emotions	Measurement	
Aghajani et al. (2021)	n = 81 (–) CD/LPE+ = 19 CD/LPE- = 31 *22% comorbid ADHD CG = 31	Forensic Community	15–19 *(M* CD/LPE ± = 16.84 *M* HC = 17.02)	LPE proxy from the Remorseless, Callousness and Unemotionality subscales of the YPI (SR) CD/LPE+ = two or more items > 5	Facial	Fear Sad	RT and accuracy (% of correct attribution)	Controlling for age and IQ, but not ADHD: 1. There were no significant differences across groups in ER as measured by RT and % of correct attribution of other’s emotions
Bennett and Kerig (2014)	n = 417 (111) Primary CU (1) = 55 Secondary CU (2) = 76 Low CU (3) = 279	Forensic	12–18 (16.5) (*M1* = 15.85 *M2* = 16.06 *M3* = 16.26)	ICU Total score (SR) CU > 26 CG < 26	Facial	Fear Anger Sad Shame Disgust	Accuracy (% of correct responses)	1. The acquired (secondary) CU group were more accurate in recognizing others disgust 2. Membership in the primary CU group was less than the acquired group as accuracy increased for recognition of disgust, but was more likely as accuracy increased for shame 3. In relation to the Low CU group, the primary CU were more accurate in the recognition of anger. There were no differences with the acquired CU group
Dadds et al. (2018)	n = 364 (102) *53% ODD/CD; 35.6% ADHD	Clinical	3–16 (8.93)	APSD/SDQ CU derived measure (Combined PR, TR, SR)	Facial	Happy Sad Anger Fear Disgust Neutral	Accuracy (Total score)	Controlling for the effects of ADHD medication: 1. Low CU traits were related with more accurate ER 2. The relationship between CU traits and ER differed according to maltreatment history; CU traits were associated with poorer recognition of those with zero or negligible history of maltreatment 3. Patterns of moderation effects of maltreatment were inconsistent across subgroups. For the group who self-reported maltreatment, CU traits were related with poor ER in those with lower anxiety 4. Both direct and moderated effects of CU on ER were not limited to fear or sadness. Negative correlations were to some extent evident for all the analyzed emotions, except happiness
De Ridder et al. (2016)	n = 55 (10)	Forensic	NR (14.8)	YPI CU subscale (SR)	Ecological	Anger Distress	Accuracy (Total score)	1. High CU adolescents were as accurate as low CU adolescents in inferring distress and anger in staff members 2. High CU adolescents notably overestimated the general intensity of both anger and distress 3. High CU adolescents perceived more anger in staff members when they were misbehaving, particularly breaking the rules
Ezpeleta et al. (2017)	n = 320 (155) CU+ /ODD+ = 51 CU+/ODD− = 24 CU−/ODD+ = 38 CU−/ODD− = 207 *8.7% comorbid ADHD (CG) = 207	Community	8 (–)	ICU Total score (TR)	Emoticons	Happy Sad Anger Fear Neutral	Accuracy RT	Controlling for sex, SES and comorbidities: 1. Children within the CU−/ODD+ and the CU+/ODD+ were less accurate than the CG in processing the information, specifically when the stimuli expressed happiness, fear or neutral 2. Children high on CU traits but low on ODD performed similarly to the CG with respect to both accuracy and RT with one exception: children high on CU traits responded faster to fear 3. Children within the CU+/ODD+ group differed in accuracy but not in RT in relation to the CG
Kahn et al. (2017)	112 (–)	Forensic	12–20 (15.5)	ICU Total score (SR)	Facial	Happy Sad Anger Disgust Fear Neutral	Accuracy	1. There were no significant main effects for CU traits or interactions with anxiety in predicting overall accuracy in the affective facial recognition task When predicting accuracy for independently identify the 6 analyzed emotions: 2. CU traits were positively associated with fear recognition accuracy at lower levels of anxiety 3. CU traits were related with poorer results in the recognition of disgust, with a trend suggesting that this relationship tend to occur at high levels of anxiety
Kimonis et al. (2016)	n = 214 (100)	At-risk Community	3–6 (4.7)	ICU Total score (24 and 12 items), Callousness and Uncaring subscales (Combined PR, TR) UNSW CU	Facial static and dynamic	Anger Fear Happy Sad Pain (only in dynamic)	Accuracy	Controlling for CP and sociodemographics: 1. Children scoring high on ICU Total and Uncaring subscale scores were less accurate in recognizing anger, fear, happiness, and sadness 2. Callousness was only associated with poor fear and sadness recognition, but not after covaring with uncaring 3. Uncaring remained significantly associated with anger, happy and sad recognition after controlling for callousness 4. There was a significant effect of age, with older children being more accurate in emotion recognition
Klapwijk et al. (2016)	n = 79 (–) ASD = 23 CD/CU+ = 23 *34.78% comorbid ADHD CG = 33	Clinical Forensic Community	12–19 (*M*ASD = 17 *M*CD/CU+ = 16.6 *M*CG = 17.1)	ICU Total score (SR) CU+ > 27 (median)	Facial	Anger Fear Neutral	Accuracy Congruency RT	1. The CD/CU+ group reacted faster than the CG 2. There were no differences across groups in the identification of other’s emotional expression 3. The CD/CU+ group reported more congruency for anger as compared to fearful emotions
Kohls et al. (2020a)	n = 1252 (796) CD = 542 (317) CG = 710 (479)	Clinical Forensic Community	9–18 (*M*CD = 14.4 *M*CG = 14.0)	YPI CU dimension (SR) ICU (PR)	Facial	Happy Sad Anger Fear Disgust Surprise	Accuracy (% correct)	1. There were higher levels of CU traits in the CD group, with no significant differences across boys and girls 2. Relative to the CG, youths with CD showed impaired emotion recognition, which was related to higher CU traits a more physical and proactive aggression 3. Elevated CU traits within the CD group were associated with overall emotion recognition impairments rather than deficits in particular emotions 3. Parent reported CU traits were not related with emotion recognition within the CD group 4.Overall, girls outperformed boys in emotion recognition
Kohls et al. (2020b)	n = 1252 (796) CD = 542 (317) CG = 710 (479)	Clinical Forensic Community	9–18 (*M*CD = 14.4 *M*CG = 14.0)	CU scale and LPE proxy from the Remorseless, Callousness and Unemotionality subscales of the YPI (SR) LPE+ = two or more items > 4	Facial	Happy Sad Anger Fear Disgust Surprise	Accuracy (% correct)	1. The subgroup of youths with CD with an emotion recognition deficit did not differ significantly from those without such deficit regarding the presence of the LPE specifier 2. Deficits in emotion processing, including emotion recognition, related to CD do not map neatly onto established DSM-5 subtypes, such as the CD+ LPE
Lui et al. (2016)	n = 103 (33)	At-risk	16–18 (16.93)	ICU Total score (SR)	Facial	Happy Sad Anger Disgust Fear Neutral	Accuracy (Total score)	1. CU traits were negatively correlated with emotion recognition 2. Emotion recognition did not mediate the association between CU traits and both affective and cognitive empathy
Martin-Key et al. (2017)	n = 77 (–) CD = 37 (CU+ = 20; CU− = 17) *46% comorbid ADHD CG = 40	Forensic Community	13–18 (*M* CD = 16.03) *M* CG = 16.20)	ICU Total score (SR) CD/CU+ > 30 (median split) CD/CU− ≤ 30	Facial dynamic	Happy Anger Sad Disgust Fear Surprise	Accuracy	1. There were no differences between CD groups in terms of ADHD 2. Participants in the CD group were less accurate than CG in the recognition of sadness, fear and disgust 3. Yet, no significant differences were found between CD/CU+ and CD/CU− groups, for any emotion 4. When CU traits were treated dimensionally, no significant correlations were found between CU traits and emotion recognition
Martin-Key et al. (2020)	n = (52) CD = 23 (CU+ = 10 CU− = 13) *13% comorbid ADHD CG = 29	Forensic Community	13–18 (*M*CD = 16.06 *M*CG = 16.22)	ICU Total score (SR) CD/CU+ > 28 (median split) CD/CU− ≤ 28	Facial dynamic	Anger Happy Sad Disgust Fear Surprise	Accuracy	1. There were no significant differences in emotion recognition between CD and CG, nor between CD/CU+ and CD/CU− groups 2. When CU traits were treated dimensionally, there were no significant correlations between CU traits in the CD group and emotion recognition for any of the six emotions
Milone et al. (2019)	n = 60 (–)	Clinical	11–17 (13.27)	APSD CU (Combined PR, SR)	Facial (eye region)	Happy Sad	Accuracy	1. The levels of CU traits were not related with abilities in emotion recognition 2. CD males high and low on CU traits did not differ in emotion recognition performance
Moore et al. (2019)	n = 1214 (656) (607 twin pairs, 251 MZ, 356 DZ)	Community	9.1–20.8 (14.1)	ICU Total score, Uncaring/Callous and Unemotional subscales (PR)	Facial	Happy Sad Anger Fear Disgust Surprise	Accuracy: Unbiased hit rate (UBHR)	1. The uncaring/callous dimension was significantly associated with impaired recognition of happiness, sadness, fear, surprise and disgust 2. The unemotional dimension was significantly associated with improved recognition of surprise and disgust 3. The total ICU score was significantly associated with impaired recognition of sadness 4. The relationship between uncaring/callousness and deficits in distress cues recognition (i.e., fear and sadness) was entirely accounted by shared genetic influences
Muñoz (2009)	n = 55 (–)	At-risk	8–16 (11.8)	ICU Total score and subscales (SR)	Facial and body posture	Happy Sad Fear Anger Surprise Disgust (only in faces)	Accuracy	1.Youths who were CU+ traits had fewer correct responses to fear and anger faces 2.Deficit in labelling fear faces and fear body postures among youths scoring highly on CU traits, although accuracy was lower for more types of emotions than fear Deficits specific to fear for CU+ 3.Boys who were poor at labelling both the fear faces and fear body postures had the highest levels of total CU traits
O’Kearney et al. (2017)	n = 124 (45) *ODD = 43 (31) CG = 17 (14) *55.4% comorbid ADHD	Clinical Community	4–8 (*M*ODD = 5.9 *M*CG = 5.9)	ICU Total score (PR) ODD/CU+ ≥ 67% (median split) ODD/CU− < 67%	Facial	Happy Sad Anger Fear	Accuracy	1.The were no significant differences between groups for emotion recognition
O’Kearney et al. (2020)	n = 74 (31) ODD = 74	Clinical	4–8 (*M*ODD = 5.9)	ICU Total score (PR)	Facial	Happy Sad Anger Fear	Accuracy	1.CU had a small negative first-order association with the ability to understand mixed emotions but not with the other emotions abilities 2. Only at lower levels of affect arousal/dysregulation there is a notable association between higher CU and poorer emotion recognition
Pauli et al. (2021)	n = 1263 (536) CD/CU+ = 248 (153) CD/CU− = 230 (130) CG = 785 (523)	Clinical Forensic Community	9–18	ICU Total score (SR) CD/CU+ ≥ 39 (median split) CD/CU− < 29	Facial	Anger Disgust Fear Happy Sad Surprise	Accuracy (% correct)	1.The CD/CU+ group performed significantly worse than de GC group, but the CD/CU− group did not differ from either group 2.Recognition accuracy was better for high-intensity than for low- intensity emotions and GC and CD/CU− groups benefited more from the increased expression intensity than the CD/CU+ 3.Group differences in emotion recognition abilities were not driven by difficulties with specific emotions such as fear and sadness 4. Female youths significantly outperformed male youths, as evidenced by a main effect of sex
Peticlerc et al. (2019)	n = 1005 (–) (504 twin pairs, 209 MZ, 295 DZ)	Community	6–7	Four items ad hoc 1. “he/she didn’t seem to feel guilty after misbehaving” 2.” he/she has been insensitive to others' feelings” 3.” his/her emotions appear superficial” 4.” has not kept his/her promises”	Facial	Happy Sad Anger Fear	Accuracy	Controlling for other problems (physical aggression, ADHD and depressive symptoms), and for the recognition of other emotions: 1.There was a relationship between CU traits, measured in kindergarten and first grade, and poor emotional recognition of fear, measured in first grade 2. The relationship between CU traits and deficits in fear recognition was genetically influenced 3. The relationship between CU traits and deficits in the recognition of sadness did not hold after controlling for other problems and the recognition of other emotions. No genetic influence was reflected in this relationship, although evidence was provided for the influence of the non-shared environment
Rehder et al. (2017)	n = 761 (657) EA = 446 CP+ CU = 20 CG = 442 AA = 315 CP+ CU = 16 CG = 291	Community at risk	6–7	ICU Empathic-prosocial and callous subscales (PR)	Facial	Anger Sad Happy Fear	Accuracy (overall and specific emotions)	Controlling for primary caregivers’ years of education and children’s age: 1.Differences among typical children (CG), children with CP-only and children with CP + CU were moderated by child race and family income for overall emotion recognition, and by child race for specific emotion recognition 2. Only among EA children, and children within families with higher income-to-needs ratio (i.e., no extreme poverty), CG showed better accuracy for overall emotion recognition than children in the CP only and CP + CU groups 3. Only among EA children, CG showed better recognition for happy faces than children in the CP only and CP + CU group 4. Children in the CP only and CP + CU groups often did not perform differently on emotion recognition accuracy
Schwenck et al. (2011)	n = 192 (–) ASD = 55 CD = 70 (CD/CU+ = 36 CD/CU− = 34) *44.8% comorbid ADHD CG = 67	Clinical	6–17 (12.3)	ICU Total score (PR) CD/CU+ ≥ 32 (median split) CD/CU− < 32	Facial morphed	Anger Happy Sad Fear Disgust	Accuracy RT	1. There were no significant differences between CD groups (CU+/CU−) in emotion recognition based on RT and the number of correctly identified emotions 2. Removing ADHD children receiving medical treatment did not change the pattern of results
Schwenck et al. (2014)	n = (64) CP = 32 (CP/CU+ = 16; CP/CU− = 16) *62.6% comorbid ADHD CG = 32	Clinical	8–16 (13.23)	ICU Total score (PR) CP/CU+ > 35.5 (median split) CP/CU− < 35.5	Facial morphed	Anger Happy Sad Fear Disgust	Accuracy RT	There were no significant differences in the analyzed variables between children with and without comorbid ADHD Regarding RT: 1. Girls with CP/CU− reacted more slowly than CG to faces developing happy, sad, and fearful expressions 2. Girls with CP/CU+ did not differ from CP/CU− and CG Regarding accuracy: 3. Girls with CP/CU+ recognized fearful expressions better than the other two groups 4. Girls with CP/CU− recognized sad expressions less often than CG 5. Girls with CP/CU+ mistook sad faces more commonly as disgusted faces than CG; whilst girls with CP/CU− identified fearful faces as disgusted more often than the CP/CU+ group
White et al. (2016)	n = 337 (185) (High LC = 107; Low LC- = 230; High PI = 96; Low PI = 241)	At-risk	3–7 (4.82)	MAP-DB Low Concern, Punishment Insensitivity (PR) High/Low groups ≥ /≤ 80th percentile	Facial	Fear Happy Anger Neutral	Accuracy Latency (mean RT)	Controlling for age, sex, temper loss, aggression, impulsivity, and the control block In terms of Accuracy: 1. The High LC group was less accurate in identifying fearful faces than the low LC; the accuracy rates did not differ for angry or happy faces 2. There were no differences between High and Low PI groups 3. Female were more accurately to emotional facial expressions than males In terms of Latency: 3. No significant differences were observed between High/Low LC and PI groups
Wolf and Muñoz (2014)	n = 50 (–)	At-risk	11–16 (14.3)	YPI CU subscale ICU Total score (SR)	Facial and Body postures Dynamic	Fear Anger Happy Disgust Sad Pain	Accuracy	Controlling for age and violent delinquency: 1. Fearful facial and bodily expressions were unrelated with CU traits 2. For the YPI-CU, there were deficits in the recognition of pain in faces, often misidentified as sadness and disgust, and anger in postures, often misidentified as happiness and disgust 3. For the ICU, results showed a significant enhancement in the recognition of anger in faces and disgust in postures
Woodworth and Waschbusch (2007)	n = 73 (14) CP = 32 CP/CU = 24 CG = 18 *84.9%* comorbid ADHD*	Clinical Community	7–12.78 (9.81)	APSD CU (Combined PR, TR) CU+ ≥ 67 (median split) CU− ≤ 63	Facial	Anger Disgust Fear Happy Sad Surprise	Accuracy	Controlling age, sex, IQ and ADHD 1. Children with higher CU scores were less accurate in labelling sad affect than children with lower CU scores 2. Children with higher CU scores were more accurate in labelling fear than children with lower CU scores, but this main effect was qualified by the CP-CU interaction trend 3. Children with high CP but low CU traits were less accurate than other children in interpreting fearful facial emotions

*AA* African American, *ADHD* attention deficit hyperactivity disorder, *APSD* antisocial process screening device, *ASD* autism spectrum disorder, *CD* conduct disorder, *CG* comparison group, *CP* conduct problems, *CU* callous-unemotional, *DZ* dizygotic, *EA* European American, *ER* emotion recognition, *GM* grandiose-manipulative, *HC* healthy control, *ICU* inventory of callous-unemotional traits, *INS* impulsive-need of stimulation, *IQ* intelligence quotient, *LC* low concern, *LPE* limited prosocial emotions, *M* media, *MAP-DB* multidimensional assessment profile of disruptive behavior, *MZ* monozygotic, *NR* not reported, *ODD* oppositional defiant disorder, *PI* punishment insensitivity, *PR* parent-reported, *RT* reaction time, *SES* socioeconomic status, *SDQ* strengths and difficulties questionnaire, *SR* self-reported, *TR* teacher-reported, *UBHR* unbiased hit rate, *UNSW* University of New South Wales, *YPI* youth psychopathic traits inventory

**Table 3:** Studies examining emotion recognition in relation to overall psychopathic traits

Study	Participants			Psychopathy measure (informant)	Emotion recognition			Main outcome specific to emotion recognition
	*N* (female)	Sample type	Age range (*M*)		Type of stimuli	Emotions	Measurement	
Blair and Coles (2000)	n = 55 (24) PSD = 11 CG = 10	Community	11–14 (12.4)	PSD Total score (TR)	Facial	Happy Surprise Fear Sad Disgust Anger	Accuracy (Total correct answers)	1. Higher scores in psychopathic traits were related with lower emotional recognition, as well as with an increased impairment in the recognition of anger, sadness and fearful expressions 2. At the dimensional level, Factor 1 (GM/CU) was inversely correlated with the ability to recognize sadness and fear. Factor 2 (INS) was inversely correlated with the ability to recognize fearful expressions 3. When comparing children high and low in psychopathic traits, results revealed a poorer recognition of sadness in the high group, controlling for mental age and sex
Blair et al. (2001)	n = 51 (-) PP = 20 CG = 31	At-risk	9–17 (*M*PP = 12.93) (*M*CG = 12.84)	PSD Total score (TR) PP > 28 CG < 20	Facial (morphed)	Happy Sad Surprise Fear Disgust Anger	Sensitivity (nº of stages needed to correctly identify the expression) Accuracy (% of errors)	1.Children with psychopathic tendencies needed significantly more stages before they could successfully recognize sad expressions 2.Children with psychopathic tendencies made more errors when processing fearful expressions, being more likely to misclassify fear as one of the other five basic emotions 3. There were no group differences for the recognition of happy, anger, disgust and surprise
Blair et al. (2005)	n = 43 (–) PP = 22 CG = 21	At-risk	11.8–15.5 (*M*PP = 13.5) (*M*CG = 12.87)	APSD Total score (TR) PP > 25 CG < 25	Vocal	Happy Disgust Anger Sad Fear	Accuracy (% errors)	1. Boys within the PP group overall made more errors than the CG 2. Boys with PP presented a selective impairment for the recognition of fearful vocal affect 3. Boys PP group did not show significant impairment in the recognition of sad vocal affect 4. When making errors on angry or disgusted expressions, children within the PP group were more likely to mistake these expressions for fearful expression 5. Within the PP group, the covariate age had a significant positive effect in the recognition of fearful affect, and the IQ had a significant effect in the recognition of fearful and disgusted vocal affects 6. At the dimensional level, there were a positive correlation between CU traits and the number of fearful and happy vocal affect recognition errors, and between GM traits and the number of fearful recognition errors. There were no significant correlations with INS
Bowen et al. (2014)	n = 100 (–) Offenders = 63 CG = 37	Forensic Community	13–17 (*M*off = 15.79) (*M*CG = 15.41)	YPI Total score and CU subscale (SR) High > 2.5 Low < 2.5	Facial (morphed)	Happy Sad Fear Anger Disgust Surprise	Accuracy (% correct recognition scores at each intensity)	1.Young offenders with high psychopathic traits were significantly worse at detecting 50% and 75% intensity of disgusted faces 2. Having high levels of psychopathic traits explained enhanced, rather than diminished, recognition of sad expressions 3. There was a positive correlation between CU traits and 25% and 100% anger recognition
Dadds et al. (2006)	Study 1 = 33 (–) Study 2 = 65 (–)	Community	Study 1 = 8–15 (12.07) Study 2 = 9–17 (13.2)	APSD/SDQ CU and AB (GM/INS) subscales (Combined PR-SR)	Facial	Happy Sad Anger Disgust Fear Neutral	Accuracy	1. AB (GM-INS) and CU traits were associated with different ER problems in young males 2. AB was uniquely associated with a tendency to over-interpret hostility in neutral faces 3. CU traits were uniquely related to poor recognition of fearful expressions, which are in part owing to visual neglect of the eye region of other people’s eyes 4. This deficit improved in the eye gaze condition, but returned in the mouth gaze condition
Fairchild et al. (2009)	n = 121 (–) EO-CD = 42 AO-CD = 39 *21% comorbid ADHD CG = 40	Community At-risk	14–18 (EO-CD = 15.8) (AO-CD = 15.5) (CG = 15.8)	YPI Total score (SR) High > 2.5	Facial (morphed)	Anger Disgust Fear Happy Sad Surprise	Accuracy (Total score)	1. Relative to CG, recognition of anger, fear, disgust, and happiness was impaired in participants with EO-CD. Also, recognition of fear was impaired in participants with AO-CD. These results were replicated when removing participants with ADHD 2. Participants with CD who were high on psychopathic traits showed impaired fear, sadness and surprise recognition, as compared to those low in psychopathic traits
Fairchild et al. (2010)	n = (55) CD = 25 *20% comorbid ADHD CG = 30	Community At-risk	14–18	YPI Total score and CU (SR) High > 2.5 in Total score	Facial (morphed)	Anger Disgust Fear Happy Sad Surprise	Accuracy (Total score)	1. Girls with CD showed impaired recognition of anger and disgust. These results were replicated when removing participants with ADHD 2. Participants with CD and high on psychopathic traits showed impaired recognition of sadness, as compared to those lower in psychopathic traits
Gillen et al. (2018)	n = 144 (49)	Forensic	11–18 (15.24)	PCL: YV subscales	Facial Vocal tone	Happy Sad Anger Fear	Accuracy (Total score)	1. The PCL: YV Total score was related to lower accuracy of happy and sad faces 2. Interpersonal (GM) traits were positively related to fearful and angry facial emotion recognition accuracy, when controlling for the other two psychopathic factors 3. Affective (CU) traits were associated with poorer accuracy in identifying happy faces and sad, angry and fearful voices 4. Behavioral (INS) traits were not related to accuracy in recognizing emotional facial or vocal tones
Kahn et al. (2016)	n = 141 (23)	Forensic	14–18 (17.03)	PCL: YV ICU SR APSD SR CPS SR YPI SR All subscales	Facial	Happy Fear Surprise Disgust Excitement	Accuracy	1. Controlling for IQ, there is no evidence of significant associations between psychopathic traits, measured by the PCL: YV, and Experiential EI 2. For self-reported measures, there was a negative association between the Callous-Disinhibited scale from the CPS and the experiential area, but it did not hold when controlling for multiple comparisons. The association was negative with the GM factor
Lemos Vasconcellos et al. (2014)	n = 41 (–) (High PP = 20 Low PP = 21)	Forensic	13–19 (*M* High PP = 16.3; *M* Low PP = 16.7)	PCL-YV Total score High PP ≥ 30 Low PP ≤ 20	Facial	Fear Sad Happy Disgust Surprise Anger	Accuracy	1. The High PP group was significantly poorer than the Low PP group in the recognition of fear in faces presented for 200 ms 2. No other differences between groups reached statistical significance; yet, effect sizes also demonstrated a moderate difference for fear recognition at 500 ms and 1 s, with worse performance for the High PP group, and small-to-moderate difference for sadness and surprise at 500 ms, with worse performance for the Low PP group
Sharp et al. (2014)	n = 417 (187)	Community	10–12 (11.33)	YPI subscales (SR)	Facial (eye region)	Happy Sad Anger Fear Disgust Surprise	Accuracy	1. GM, CU and INS significantly (and inversely) correlated with emotion recognition 2. When the three dimensions were modeled together in a regression framework, only CU traits significantly predicted emotion recognition, but only in relation to complex emotions 3. No psychopathy dimension were predictive of basic emotion recognition
Stevens et al. (2001)	n = 18 (–) (PSD+ = 9; PSD− = 9)	At-risk	9–15 (11.7)	PSD Total score (TR) PSD+ > 25 PSD− < 20	Facial Auditory	Happy Sad Anger Fear	Accuracy	1. The PSD+ group was less likely to name the facial or vocal affect correctly than the PSD- group 2. Both groups were significantly more likely to name the facial expressions than the vocal affects 3. The PSD+ group showed selective impairments in the recognition of both sad and fearful facial expressions and sad vocal tone 4. The two groups did not differ in their recognition of happy of angry facial expressions, and fearful, happy and angry vocal tones
Sylvers et al. (2011)	n = 88 (–)	At-risk	7–11 (8.88)	APSD subscales (Combined PR-SR)	Facial	Neutral Happy Fear Disgust	RT Accuracy of face location	1. Children with high CU scores exhibit preattentive or automatic preconscious fear-recognition deficits (large effect) and disgust recognition deficits (moderate effects) 2. CU traits provided an incremental contribution in predicting preattentive fear-recognition deficits (large effect) and disgust recognition deficits (moderate) above and beyond GM and INS traits 3. CU traits interacted with INS traits (moderate effect) in predicting preattentive fear-recognition deficits, with relation between CU traits and preattentive fear-recognition increasing as INS traits increased

*AB* antisocial behavior, *ADHD* attention deficit hyperactivity disorder, *AO-CD* adolescence-onset conduct disorder, *APSD* antisocial process screening device, *CD* conduct disorder, *CG* comparison group, *CPS* child psychopathy scale, *CU* callous-unemotional, *DBD* disruptive behavior disorder, *EI* emotional intelligence, *EO-CD* early onset conduct disorder, *ER* emotion recognition, *GM* grandiose-manipulative, *INS* impulsive-need of stimulation, *IQ* intelligence quotient, *LPE* limited prosocial emotions, *M* media, *NR* not reported, *PCL* YV psychopathy checklist: youth version, *PP* psychopathic traits/tendencies, *PR* parent-reported, *PSD* psychopathy screening device, *RT* reaction time, *SDQ* strengths and difficulties questionnaire, *SR* self-reported, *TR* teacher-reported, *YPI* youth psychopathic traits inventory

**Table 4:** Studies examining attention biases (AB) in emotion recognition (ER) in relation to CU/psychopathic traits

Study	Participants			CU/psychopathy measure (informant)	Emotion Recognition			Attention biases		Main outcome specific to attention biases and emotion recognition
	*N* (female)	Sample type	Age range (*M*)		Type of stimuli	Emotion	Measurement	AOI	Measurement	
Bedford et al. (2017)	n = 206 (~ 105)	Community	Up to 7 years (*M*T1 = 6 m) (*M*T2 = 2.5) (*M*T3 = 6) (*M*T4 = 7)	ICU Total score (PR)	Facial	Happy Sad Mad Scared	Accuracy (Total score of correct/incorrect answers)	Face (not defined)	Proportion of the total valid interaction time	1. Poorer ER at age 6 predicted higher CU trait at age 7, controlling for earlier CU traits (age 2.5) 2. Infants with low levels of gaze to the parent’s face showed higher CU traits at age 7, only when maternal sensitivity was low 3. Infant attention to the face was not related with later ER 4. ER appears to act as an independent predictor of later CU traits, rather than mediating their association with infant’s mother-directed gaze and maternal sensitivity
Billeci et al. (2019)	n = 58 (–) CD = 16 ODD = 19 CG (HC) = 23	Clinical	7–10 *(M*DBD = 8.93) *(M*HC = 8.86)	APSD CU subscale (Combined PR, TR)	Facial	Happy Sad Anger Fear Disgust Neutral	Accuracy	Face Eyes Mouth	Number of fixations Average length of fixations Length of first fixation	1. In the total sample, higher levels of CU were associated with a poorer recognition of sadness, and lower Nº fixations and Average length of fixations on the eye region of sad faces, even after controlling for covariates 2. In children with a DBD diagnosis, high levels of CU traits were associated with lower Average length of fixations on the eye areas of sad faces, which in turn predicts lower levels of sadness recognition (mediation process)
Bours et al. (2018)	n = 122 (–) ASD = 50 ODD/CD = 44 *41% comorbid ADHD CG = 28	Clinical	12–19 (15.4)	ICU Total score (PR, SR) YPI Total score and CU subscale (SR)	Facial	Happy Sad Anger Fear Neutral	Accuracy (% correct answers)	Face Eyes Mouth	Total fixation duration Time to first fixation % Total fixation	1. Higher scores in psychopathic traits within the ODD/CD group were related with a lower Time to first fixation to the eyes for the recognition of fear. This association did not survive when correcting for multiple comparisons 2. Sensitivity analyses revealed that results were not influenced by the presence of comorbid ADHD or by medication use
Dadds et al. (2008)	n = 100 (–)	Community	8–15 (12.4)	APSD/SDQ CU and AB (GM/INS) factors (Combined PR-SR) Low < 25th High > 75th	Facial	Happy Sad Anger Disgust Fear Neutral	Accuracy (Total score)	Free Eyes Mouth	Primacy Number & Duration of fixation	1. High CU traits were associated with poorer fear recognition 2. High CU traits were related with lower number and duration of eye fixations, and fewer first foci to eye region, which would account for the deficit in recognizing fear 3. These results were not affected by the inclusion of antisocial problems (GM/INS), emotional problems, hyperactivity and peer problems as covariates 4. These deficits were no longer evident under the eye gaze condition, especially the number of times that subjects looked at the eyes first
Dadds et al. (2011)	n = 92 (–)	Clinical	5–16 (8.93)	APSD/SDQ CU and AB (GM/INS) subscale (Combined PR, TR, SR) High/low = combined 50th	Facial	Happy Sad Anger Fear Disgust Neutral	Accuracy (Total score of correct fear recognition)	Eyes	% Number of times eye contact was made divided by the number of intervals in which the dyad interacted	1. Males high on CU traits showed consistent impairment in eye contact towards their parents 2. Fathers of high CU boys showed less eye contact with their child. Mothers of CU boys did not show impairments 3. Levels of eye contact were related with independent measures of fear recognition 4. Increased levels of eye contact in child-father and father-child dyads were related with increased recognition of fear 5.Levels of antisocial behavior (GM/INS), hyperactivity, anxiety/depression, peer problems, and autism type did not influence the results
Demetriou and Fanti (2022)	n = 59 (27) CU+ = 31 (14) CU− (CG) = 28 (13)	Community	5–10 (7.5)	CPTI CU subscale (PR: mothers & fathers) CU+ ≥ 1SD CU− ≤ 1SD	Facial	Happy Fear Sad Anger	Accuracy (Sum of missclassifications)	Eyes Mouth	Total fixation duration	1. High CU children (CU +) made more accuracy errors, irrespective of the emotion expressed, compared to their CU− counterparts 2. High CU children showed lower rates of total fixation duration in the eye region for all the emotional expressions in both adult and peer pictures 3. High CU children showed an increased concentration to the mouth area compared to the CU− children, suggesting an ineffective pattern of processing emotions
Hartmann and Schwenck (2020)	n = 94 (36) ODD = 29 (7) CD = 1 (1) *29% comorbid ADHD CG = 65 (30)	Clinical Community	8–14 (10.4)	ICU Total score (PR)	Facial	Anger Sad Fear	Paradigm 1: Accuracy (error rates) RT Paradigm 2: Accuracy (error rates)	Eyes Mouth	Fixation count Total fixation duration	Paradigm 1: Recognition 1. Independent of CP (including ADHD), age and gender, CU traits were related with slower recognition of angry, sad and fearful facial expressions, but not with higher error rates Paradigm 2: Categorization 2. Higher levels of CU traits significantly predicted greater number of errors over all emotions 3. Higher levels of CU traits were related with higher mistakes during the anger trials, only when CP were high 4. Higher levels of CU traits predicted the number of mistakes in fear trials in which only the eyes were presented, but only when CP were low 5. Higher levels of CU traits predicted more mistakes in anger trials when only the eyes were presented. High levels of CP predicted low mistakes 6. High levels of CU traits predicted more mistakes in the sad trials when only the mouth of the stimuli was presented 7. There was no evidence that the association between CU traits and emotion processing could be explained by misguided attention
Levantini et al., (2022a)	n = 116 (–) ODD = 94 CD = 22 *48.28% comorbid ADHD	Clinical	7 –12 (9.0)	APSD subscales (Combined PR, TR)	Facial	Happy Sadness Anger Disgust Fear Neutral	Accuracy	Face Eyes Mouth	Number of fixations (FC), Average length of each fixation (FD), Length of first fixation (FFD)	Controlling for age, IQ, externalizing problems and SES: 1. CU traits were significantly and negatively associated with sadness recognition and narcissism was negatively associated with disgust recognition 2.Regarding gaze pattern, CU traits were negatively associated with length or first fixation to the mouth of angry faces, number of fixations to the eyes of sad faces and length of first fixation to the eyes of disgusted faces 3. Impulsivity (INS) was positively associated with the number of fixations and average length of each fixation to the eyes of angry faces and number of fixations to the eyes of fearful faces 4. Narcissism (GM) was negatively associated with the number of fixations to the eyes of angry faces, and positively associated with the number of fixations to the mouth of angry faces
Martin-Key et al. (2018)	n = 101 (49) CD = 50 (24) *34% comorbid ADHD CG = 51 (25)	Forensic Community	13–18 *M*CD_male_ = 15.94) *M*CD_female_ = 16.21) *M*CG_male_ = 16.22) *M*CG_female_ = 16.40)	ICU Total score (SR)	Facial static and dynamic	Anger Sad Fear Happy Surprise Disgust Neutral	Accuracy	Eyes Mouth	Initial eye preference Total eye preference	Controlling for subject, comorbidity and age: 1. Higher levels of CU traits were associated with poorer fear recognition across the whole sample 2. Within the CD group, those high on CU traits showed better fear recognition, and reduced attention to the eyes for surprise faces 3. CU and emotional intensity interacted to predict initial eye preference for surprise, which increased with emotional intensity and was larger for high CU participants 4.With exception of disgust, females showed greater total eye preferences than male for all emotions
Martin-Key et al. (2021)	n = 96 (48) CD = 45 (23) *20% comorbid ADHD CG = 51 (25)	Forensic Community	13–18 *M*CD_male_ = 15.80) *M*CD_female_ = 16.36) *M*CG_male_ = 16.22) *M*CG_female_ = 16.40)	ICU Total score (SR)	Body postures (dynamic and static)	Anger Fear Neutral	Accuracy	Arms	Arm preference score (% of time fixation)	Controlling for subject, age and psychiatric comorbidity: 1. There were no effects of CU traits on body posture recognition 2. The effects of CU traits varied according to CD status and sex, with CD males with lower levels of CU traits showing the most atypical fixation behavior More specifically: 3. Higher levels of CU traits predicted higher arm preference score across the entire sample, yet, CU traits were negatively associated with arm preference scores in females, but positively associated in males 4. For fearful and neutral body postures, CU traits were related with arm preference scores in the CD group. These association were negative for females, but positive for males (in the total sample) 5. This atypical fixation behavior did not explain the body posture recognition deficits observed in CD
Muñoz et al. (2021)	n = 73 (12) *52% *ADHD*	Clinical	11–16 (14.0)	ICU Total score and subscales APSD I/CP score (SR)	Facial	Fear Anger Happy Neutral	Accuracy	Eyes Mouth	Reflexive attentional orienting (number, direction and velocity of saccades)	1.Children high on CU traits did not show a significant deficit in reflexive gaze to the eye region of fearful faces 2. Similar non-significant effects were observed for the other emotions 3. Children high on CU traits performed more poorly in labelling fearful faces accurately, only when the mouth region and not the eye region of the face was primed. Yet, there were no significant differences regarding the association between ICU scores and fear recognition across the mouth-fixation and the eye-fixation condition 4. Youths high on I/CP who are also high on CU traits shifted their gaze less toward fearful eyes when initially focused on the mouth. Only the Callousness facet was significantly associated with decreased gaze shift

*AB* antisocial behavior, *ADHD* attention deficit hyperactivity disorder, *AOI* area of interest, *APSD* antisocial process screening device, *ASD* autism spectrum disorder, *CD* conduct disorder, *CG* comparison group, *CP* conduct problem, *CU* callous-unemotional, *CPTI* child problematic traits inventory, *DBD* disruptive behavior disorder, *ER* emotion recognition, *FC* fixation count, *FD* fixation durations, *FFD* first fixation duration, *GM* grandiose-manipulative, *HC* healthy control, *I/CP* impulsivity/conduct problems, *ICU* inventory of callous-unemotional traits, *INS* impulsive-need of stimulation, *NR* not reported, *M* media, *ODD* oppositional defiant disorder, *PR* parent-reported, *RT* reaction time, *SD* statistic deviation, *SDQ* strengths and difficulties questionnaire, *SR* self-reported, *TR* teachers-reported

A narrative approach was used to synthesize the findings for each study, obtained by compiling the information from the extraction sheet form. A minimum of five studies reporting similar results was used as a criterion to extract firm conclusions. Nevertheless, inconsistent findings or descriptive results based on a smaller number of studies were also reported as they may provide relevant information to establish new ways of discussion and analysis in future research.

## Results

### Study Selection

**Fig. 1:**
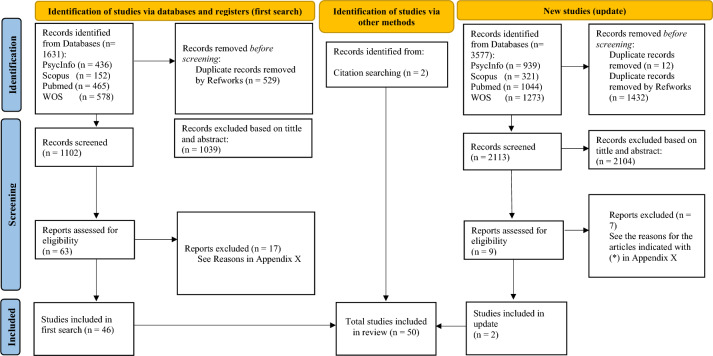
Flowchart for systematic review process. Adapted from the flow diagram of PRISMA (Page et al., 2021)

The systematic search was conducted in two different time points; the first one provided 1631 titles from the electronic database search, and two additional references located via reference-list searches. The second one (update) provided 3577 titles. After excluding duplicates, 4679 studies were screened based on titles and abstracts. A total of 76 articles were selected for full-text assessment of eligibility, and the remaining articles were excluded for being off-topic and because they failed to meet the minimum inclusion criteria for this systematic review. After the full text assessment, 50 articles were finally included in the review. The excluded articles and the reasons for their exclusion are available in Supplementary Material (Table S5). The entire selection process is represented in the flowchart of Fig. 1.

The inter-rater agreement Cohen’s Kappa revealed an almost perfect agreement (K = 0.89) between the researchers regarding the decisions to include or exclude the eligible studies.

### Study Characteristics

Table S3, available online, provides a summary of the main characteristics of each included study. The 50 included studies were published between 2000 and 2022 and provide data from 12,139 children and adolescents (40.84% females [N = 4958]; *M* age = 12.27) belonging to clinical (*k* = 10), forensic (*k* = 6), community (*k* = 9), at-risk (*k* = 9) and combined (*k* = 16) samples. Depending on age, we can distinguish between samples of children (i.e., up to 12 years old; *k* = 13), samples of adolescents (i.e., from 12 years old;* k* = 16), and mixed samples of children and adolescents (*k* = 20). Within children’s samples, the majority of the analyzed articles have focused on middle childhood (*k* = 9). A few articles included combined samples from early and middle childhood (*k* = 3). Only one study examined emotion recognition deficits in preschoolers (Kimonis et al., 2016). Most of the population analyzed came from European samples (*k* = 26; 11 UK; 3 Germany; 3 Netherlands; 2 Cyprus; 2 Italy; 1 Spain; 1 Switzerland and five with no region specified). The next most frequent location was USA (*k* = 9), Australia (*k* = 4), Canada (*k* = 2) and Brazil (only 1 study). Ethnicity was reported in 27 studies being White/Caucasian (*k* = 19) and African American (*k* = 7) the most analyzed ethnic groups.

The most common diagnosis notified was CD (*k* = 16), followed by ODD (*k* = 7) and CP (*k* = 3). In some cases, the clinical sample was classified according to the level of CU traits (Bennet & Kerig, 2014; Demetriu & Fanti, 2022), the specifier with Limited Prosocial Emotions (LPE), under the label “offenders” (Bowen et al., 2014), based on levels of Low concern (LC) and punishment insensitivity (PI) (White et al., 2016), or based on the presence/absence of “psychopathic personality” (Blair et al., 2001, 2005; Lemos Vasconcellos et al., 2014).

Regarding the clinical samples (i.e., children and adolescents with (sub)clinical levels of CP, ODD and CD) (n = 2724; 22.44% of total sample), some articles reported the number of comorbid ADHD (n = 2724; 24.63% of clinical samples). Also, despite not being considered in this review, in three cases ASD was notified (Bours et al., 2018; Klapwijk et al., 2016; Schwenck et al., 2011). All this information was added to the descriptive sample section, in all results tables (see Tables 2, 3, 4).

The presence and intensity of psychopathic traits have been assessed with various instruments, including the Youth Psychopathic Traits Inventory (YPI; Andershed et al., 2002) (*k* = 9), the Inventory of Callous Unemotional Traits (ICU; Frick, 2004) (*k* = 24; 48%), the Antisocial Process Screening Device (APSD; Frick y Hare, 2002) (*k* = 12), the Psychopathy Screening Device (PSD; Frick et al., 1994) (*k* = 3), the Strengths and Difficulties Questionnaire (SDQ; Goodman, 1997) (*k* = 4), the Child Problematic Traits Inventory (CPTI; Colins et al., 2014) (*k* = 1), the Psychopathy Checklist: Youth Version (PCL: YV; Forth et al., 2003) (*k* = 3), Items ad hoc (*k* = 1) and the Multidimensional Assessment Profile of Disruptive Behaviour (MAP-DB; Wakschlag et al., 2012) (*k* = 1). Sometimes cut-off points were used to determine the presence or absence of psychopathic traits (especially CU traits), which have been arbitrarily chosen according to different studies. Regarding CU traits, sometimes the subdivision in three dimensions (i.e., callouness, uncaring, and unemotional) was also considered.

The affective factor of psychopathy (i.e., CU traits) has been the most evaluated psychopathy dimension (*k* = 33; 66%). Studies that just focused on CU traits are described in Table 2. These traits can be found in different degrees of intensity (high/low) or can be conceptualized dichotomously (presence/absence). Also, 17 studies considered psychopathic traits as a multidimensional construct. Most of them (*k* = 9) considered psychopathic personality as a composite total score, with 3 also providing data on specific CU traits (Bours et al., 2018; Bowen et al., 2014; Fairchild et al., 2010). The remaining studies focused on different subdimensions (i.e., GM, CU, INS) (*k* = 5), or used alternative combinations (e.g., GM-INS, CU) (Dadds et al., 2006, 2008, 2011). Articles assessing psychopathic traits from a multidimensional perspective are reported in Table 3.

Considering design, 24 studies compared performance in emotion recognition across the whole sample using a correlational design. In the remaining (*k* = 26), a between-group design was used. To determine membership of the comparison group, one of the following criteria was considered: CU levels (high vs. low), cut-off points based on psychopathy assessment instruments, no clinical disorders and not being an offender. Most studies were cross-sectional (*k* = 45; 90%), whilst just five studies provided longitudinal results (Bedford et al., 2017; De Ridder et al., 2016; Peticlerc et al., 2019; Rehder et al., 2017).

All studies included in the present review employed an emotional recognition task. Most of the articles examined the recognition of basic emotions (Ekman, 1992). However, the most repeated emotions were distress emotions (fear and sadness). Fear was analyzed in all articles except 1 (98%) (Milone et al., 2019), whilst sadness was considered in 44 articles (88%). Some articles assessed more emotions than the basic ones (i.e., excitement, shame, pain, mad, scared) (Bedford et al., 2017; Bennett & Kerig, 2014; Kimonis et al., 2016), but these results were not considered for the purpose of the current review.

The most frequent emotion recognition tasks used were UNSW Facial Emotion Task (*k* = 6), The Emotion Hexagon Task (*k* = 5), NimStim Set of Facial Expressions (*k* = 4), Facial Emotion Recognition Task (*k* = 3), RaFD (*k* = 3), DANVA-II (*k* = 2) y CET (*k* = 2). Both accuracy and reaction time were the most used measures for emotion recognition. The emotion recognition task was sometimes complemented by another task to assess attentional bias (*k* = 11). The characteristics of the attentional tasks, with their specific results, are presented in Table 4. Only two of these articles examined attentional bias by assessing gaze direction between mothers and children (Bedford et al., 2017; Dadds et al., 2011). In the others, eye tracker devices were used. To assess the attentional pattern, AOI were established: eyes and mouth (*k* = 8), only the face (Bedford et al., 2017), eyes (Dadds et al., 2011) or arms (Martin-Key et al., 2021). The analysis of the response considered accuracy, reaction time (RT), number and duration of fixations.

The most analyzed stimulus type was facial and human (*k* = 43; 86%). Bodily stimuli (Martin-Key et al., 2021; Muñoz, 2009; Wolf & Muñoz, 2014) and vocal tones were also examined (Blair et al., 2005; Gillen et al., 2018; Stevens et al., 2001), as well as non-human stimuli, including doll faces (O’Kearney et al., 2017, 2020) and emoticons (Ezpeleta et al., 2017).

Stimuli presentation time ranged from 200 ms (Lemos Vasconcellos et al., 2014) to 6 s for static images (Bours et al., 2018), and from 1 s (Kimonis et al., 2016; Martin-Key et al., 2018) to 9 s (Schwenck et al., 2011, 2014) for dynamic emotional stimuli. In the case of video clips, the presentation ranged between 3 s (Martin-Key et al., 2021) and 144 s (Martin-Key et al., 2017, 2020).

### Risk of Bias in Studies

The evaluation made from AXIS and CASP suggests overall a low to moderate level of bias among the eligible studies (see Tables S4, S5, available online). Most of the included studies provided good indicators of quality, suggesting a low risk of bias. Exceptionally, some articles that did not provide sufficient information for replicability (Milone et al., 2019) or used subjective measures that may introduce considerable bias into the study (De Ridder et al., 2016).

### Results of Individual Studies

The main results obtained in the articles included in this systematic review are reported in Tables 2, 3 and 4. The first two analyze those studies that consider CU traits (Table 2) and all psychopathic traits (Table 3) respectively. The third one (Table 4), groups together all those articles that evaluated attentional bias in addition to emotion recognition.

#### Emotion Recognition and Psychopathic Traits

##### CU Traits

CU traits was the most studied dimension (*k* = 33). Evidence was found on the relationship between high levels of CU traits and a deficit in *general emotion recognition* (Bedford et al., 2017; Demetriou & Fanti, 2022; Hartmann & Schwenck, 2020; Lui et al., 2016). In addition, this impairment also affected the recognition of some *specific emotions.* Hence, Woodworth and Waschbusch (2007) found that children high on CU traits were less accurate in labelling sadness. Fear recognition has also been affected both in isolation (Dadds et al., 2008; Martin-Key et al., 2018; Muñoz, 2009; Peticlerc et al., 2019; White et al., 2016) and in combination with other distress emotions, including anger (Muñoz, 2009), disgust (Sylvers et al., 2011) and sadness (Billeci et al., 2019).

It seems that emotional complexity may play a role in emotion recognition, as postulated by some authors (Adolphs & Tranel, 2004). In this sense, Sharp et al. (2014) found that CU traits might imply a difficulty in the recognition of complex emotions as opposed to simple emotions. Moreover, this deficit seems to affect all modalities: bodily (Muñoz, 2009), vocal (fear and happy; Blair et al., 2005; sad, angry and fear; Gillen et al., 2018) and facial (all other studies reviewed in this section). Evidence was also found in regards emotional processing, measured as reaction time, with children high on CU traits recognizing the emotions of anger, sadness and fear more slowly (Hartmann & Schwenck, 2020).

In some cases, no significant differences according to the level of CU traits were found in the accuracy of recognizing distress emotions, including angry, sad and fearful faces (Hartmann & Schwenck, 2020), also in ecological environments (De Ridder et al., 2016). In this ecological assessment, conducted in a forensic sample, CU participants seemed to overestimate the intensity of distress and anger in staff members, particularly when they were misbehaving (De Ridder et al., 2016). In regular task conditions, the lack of relationship between CU traits and recognition of fearful faces and body postures varied depending on the assessment instrument used (Wolf & Muñoz, 2014). A similar result was also found in Bowen et al. (2014), with a positive correlation between CU traits and 25% and 100% anger intensity recognition.

In sum, there was a broad trend to relate CU traits with moderate pervasive emotion recognition (Lui et al., 2016) as well as a specific deficit for fear, with effect sizes ranging from moderate (Martin-Key et al., 2017) to large (De Ridder et al., 2016; Woodworth & Waschbusch, 2007). Moderate (Martin-Key et al., 2017) and large effects were also found for sadness (De Ridder et al., 2016). These deficits transcended modality and type of stimulus presented. Emotion processing was also strongly impaired, with longer time required for emotional recognition in children and adolescents high on CU traits.

##### CU Subdimensions

Two studies considered the role of subdimensions of CU traits, with some mixed results even in effect sizes, which have consistently been small for the different subdimensions (Kimonis et al., 2016; Moore et al., 2019) and have ranged from small (Moore et al., 2019) to moderate for the total ICU score (Kimonis et al., 2016). On the one hand, Kimonis et al. (2016) found that scoring high on the ICU total score was associated with lower accuracy in recognizing fear, anger, happiness and sadness. Regarding subdimensions, callousness was only associated with poor fear and sadness recognition but not after controlling for uncaring. Moreover, uncaring remained significantly associated with anger, happy and sad recognition after controlling for callousness. On the other hand, Moore et al. (2019) found that the ICU total score was significantly associated with impaired sadness recognition. Furthermore, the uncaring/callousness subdimension was significantly associated with impaired recognition of happiness, sadness, fear, surprise and disgust. For all these emotions, the relationship between uncaring/callousness and the recognition of distress emotions was entirely accounted by shared genetic influences. An opposite pattern of results was observed for the unemotional subdimension, which was significantly associated with improved recognition of surprise and disgust.

##### CU Variants

Some scholars pointed to the existence of two CU variants. Primary callousness arises as a function of a genetically based deficit in emotion processing mainly characterized by a lack of emotional distress and anxiety (Blair et al., 2006). In contrast, acquired callousness proposes that CU might arise trough the result of environmental factors, with anxiety playing a central role in the definition of the secondary variant (Kerig & Becker, 2010). Following these premises, Bennett and Kerig (2014) investigated the importance of these two variants in emotion recognition, finding differences depending on the type of CU variant. Thus, the acquired or secondary group were more accurate in recognizing others disgust whilst primary CU were more accurate in the recognition of anger compared with the low CU group. For both primary and secondary variants, the relations with emotion recognition were strong. In addition, the ability to recognize certain emotions determined the classification of the adolescents into the primary (more accuracy for shame) or secondary group (more accuracy for disgust).

##### Psychopathic Traits (GM, CU, INS)

Higher scores in psychopathic traits, measured as a multidimensional construct, were related with lower facial emotional recognition (Blair & Coles, 2000; Dadds et al., 2006; Sharp et al., 2014; Stevens et al., 2001), as well as with an increased impairment in the recognition of happy (Gillen et al., 2018) anger (Blair & Coles, 2000), sadness (Blair & Coles, 2000; Gillen et al., 2018; Stevens et al., 2001) and fearful expressions (Blair & Coles, 2000; Lemos Vasconcellos et al., 2014; Stevens et al., 2001). Moreover, when comparing groups with high and low levels of psychopathic traits, results revealed a poorer recognition of sadness in the high psychopathic traits (PP) group.

Processing (RT) and recognition (accuracy) were also impaired for distress emotions (i.e., sadness and fear), with children with psychopathic traits, who need more stages (i.e., emotional complexity) to recognize disgust (Bowen et al., 2014) and sad expressions (Blair et al., 2001). Also, they made more errors with fear expressions, being more likely to misclassify fear as one of the other five basic emotions (Blair et al., 2001), and more specifically with angry expressions (Blair et al., 2005).

These deficits were seen in the same direction when vocal stimuli were considered (Blair et al., 2005; Stevens et al., 2001). In this sense, boys with psychopathic traits presented a selective impairment for the recognition of fearful vocal affect (Blair et al., 2005) or were less accurate to correctly identify the sad vocal affect (Stevens et al., 2001). One of the included studies examined emotion recognition as part of emotional intelligence and found no relationship between experiential emotional intelligence, measured as a proxy of emotion recognition through facial emotion assessment, and the combination of the three psychopathy dimensions (Kahn et al., 2016).

Overall, as was observed for CU traits, the presence of psychopathic traits entailed a lower facial and vocal emotion recognition focused on distress emotions (fear and sadness), with effect sizes ranging from moderate (Gillen et al., 2018; Lemos Vasconcellos et al., 2014) to large (Blair & Coles, 2000) for both emotions and modalities. Moreover, processing was also significantly impaired, needing more time to react or more stages of each morphed emotion to reach an accurate identification.

##### Other Combinations of Psychopathic Traits

At the dimensional level, GM/CU traits, representing the Factor 1 of psychopathic personality, were moderately inversely correlated with the ability to recognize sadness and fear (Blair & Coles, 2000). Yet, in Blair et al. (2005) no significant correlation was found. GM- INS were associated with different emotion recognition problems (Dadds et al., 2006). Considering psychopathic traits separately, INS was inversely correlated with the ability to recognize fearful expressions (Blair & Coles, 2000) or not related to accuracy in recognizing emotional facial or vocal tones (Gillen et al., 2018). In other studies, INS interacted with CU traits in predicting preattentive fear-recognition deficits (Sylvers et al., 2011). In the case of GM, this psychopathic dimension affects the number of fearful recognition errors (Blair et al., 2005; Gillen et al., 2018) and the accuracy to identify angry facial emotions (Gillen et al., 2018).

#### Measurement of Psychopathic Traits

Several different assessment instruments have been used for the measurement of psychopathic traits (see “Study Characteristics” section). Particularly noticeable is the variability when considering the informant [parent-report (PR), teacher-report (TR) and self-report (SR)], the focus on different subscales (e.g., total scores, CU traits), the dimensional versus categorical conceptualization of the variables, and the cut-off points employed to define high/low scores on the intended measures, even when using the same instrument. This heterogeneity can be observed in Tables 2, 3, 4, included in the text.

The **Inventory of Callous Unemotional Traits** (ICU; Frick, 2004) (*k* = 24; 48%) was the most widely used. When the *informant was the child/ adolescent* (i.e., SR) and the total score of the instrument was considered, most of the reviewed studies did not find deficits associated with CU traits and emotion recognition (Klapwijk et al., 2016) or attention biases (Bours et al., 2018; Martin-Key et al., 2021; Muñoz et al., 2021), even when CU traits were treated dimensionally (Martin-Key et al., 2017, 2020). When deficits in emotion recognition were reported, they tended to be more pervasive (e.g., Pauli et al., 2021) rather than specific (e.g., fear; Muñoz, 2009), and particularly in at risk samples (Lui et al., 2016). These deficits were typically associated with other variables examined in the study, including CU variants or anxiety (Bennett & Kerig, 2014; Kahn et al., 2017), or were linked to sample characteristics (e.g., clinical groups; Martin-Key et al., 2018). If we consider *external informants* (i.e. parents and teachers), the variability of the results continues to be maintained, with a certain tendency towards an appreciation of deficits in the recognition of sadness (Hartmann & Schwenck, 2020; Moore et al., 2019; Schwenck et al., 2014), mixed emotions (O’Kearney et al., 2020), and happy, fearful, neutral and angry facial expression (Ezpeleta et al., 2017; Hartmann & Schwenck, 2020). Also, a relation between poorer emotion recognition and high CU was found (Bedford et al., 2017). Only two studies found no relationship between CU traits and emotional perception (O’Kearney et al., 2017) or recognition (Schwenck et al., 2011), when using parent’s or teacher’s reports. When multiple subscales were used (i.e., Callousness, Uncaring, Unemotional), both impaired and enhanced emotion recognition were found (Kimonis et al., 2016; Moore et al., 2019; Rehder et al., 2017). As is the case of the self-reported measures, the outcomes sometimes depended on the moderator variables, including sex (Schwenck et al., 2014) and ethnicity (Rehder et al., 2017).

When using the **Antisocial Process Screening Device** (APSD; Frick y Hare, 2002) (*k* = 12) all but one study (Muñoz et al., 2021), showed deficits in emotion recognition, especially for fear (Woodworth & Waschbusch, 2007) and sadness (Dadds et al., 2006, 2008), as well as an aberrant pattern in attention, especially to the eyes (Billeci et al., 2019; Dadds et al., 2008, 2011). It is important to note that deficits in emotion recognition were mostly observed with the APSD total score, considering all the psychopathy dimensions, irrespectively of the informant (Blair & Coles, 2000; Blair et al., 2001, 2005; Dadds et al., 2006, 2008; Levantini et al., 2022a; Sylvers et al., 2011).

For **The Youth Psychopathic Traits Inventory** (YPI; Andershed et al., 2002) (*k* = 9), which is a self-reported measure, all studies found deficits in multiple emotions, i.e., disgust (Bowen et al., 2014), sadness (Fairchild et al., 2009, 2010) and complex emotions (Sharp et al., 2014), across all psychopathy dimensions. Again, results differed in relation to potential moderators, as sample type (e.g., offenders; Bowen et al., 2014) or the use of specific subscales (e.g., YPI CU; De Ridder et al., 2016).

In some cases, various instruments were simultaneously used to measure psychopathic traits. Separate results for the measurements employed were sometimes not provided (Bours et al., 2018; Dadds et al., 2008, 2011, 2018; Kahn et al., 2016; Kohls et al., 2020a). In other cases, studies reported different results according to the instrument (Kahn et al., 2016; Wolf & Muñoz, 2014). As an example, Wolf and Muñoz (2014) showed that CU traits measured through the ICU were related with a significant enhancement in the recognition of anger faces and disgusted body postures, a result that did not replicate when using the CU measure of the YPI. Finally, some studies showed differences across instruments that seemed more related with the dimension assessed (e.g., ICU total versus callousness) rather than the instrument itself (Kimonis et al., 2016; Muñoz et al., 2021).

In sum, as previously observed, variability in the results is the dominant trend for both accuracy and attention. Nevertheless, we can identify certain patterns. Potential moderator variables examined in the review (e.g., age, gender, sample type) seemed to play a significant role in determining deficits in emotion recognition. In the case of CU traits measured with the ICU, more specific deficits were observed when data was reported by parents and/or teachers rather than self-reports. Finally, the use of different subscales, combining various assessment instruments, and employing different cut-off points could have affected the results, increasing their variability.

#### (Sub)Clinical Groups: The Role of Disruptive Behavior

Expectedly, higher levels of psychopathic traits were reported within the groups of children and adolescents with CP, or with the clinical groups (CD/ODD) (Kohls et al., 2020a; Martin-Key et al., 2018, 2020). CD was the clinical condition most studied (*k* = 13). Within this group, when we consider high levels of psychopathic traits, results showed deficits in fear, sadness and surprise emotions (Fairchild et al., 2009), as well as a unique deficit in recognizing sadness (Fairchild et al., 2010) in both male and female samples respectively. However, results vary when we only analyze CU traits. Thus, even though one study showed that elevated CU traits within the CD group were associated with overall emotion recognition impairments, rather than deficits in particular emotions (Kohls et al., 2020a), in most of the examined studies no significant differences were found, regardless of the type of stimulus used, as a function of CU traits (Aghajani et al., 2021; Kohls et al., 2020a; Kohls et al., 2020b; Klapwijk et al., 2016; Martin-Key et al., 2017, 2020, 2021; Milone et al., 2019; Schwenck et al., 2011). Indeed, in all of the aforementioned studies but two (Aghajani et al., 2021; Martin-Key et al., 2020) deficits in emotion recognition were uniquely related with the presence of CD. In addition to this finding, some authors pointed out that this group (CD/CU+) would not benefit from increasing the emotional intensity of the stimuli to enhance recognition (Pauli et al., 2021). Regarding processing, higher levels of CU traits indicated faster processing speed, requiring less time to recognize emotions in general (Klapwijk et al., 2016) and fear in particular (Martin-Key et al., 2018).

This pattern of mixed results was also replicated if we analyze the clinical condition constituted by *ODD* (*k* = 3). In this regard, it was found that (a) high or low levels of CU traits did not affect emotional recognition (Ezpeleta et al., 2017; O’Kearney et al., 2017), (b) only affected the recognition of mixed emotions (O’Kearney et al., 2020), or (c) even higher levels of CU traits were associated with better recognition of fear (Ezpeleta et al., 2017).

Within the group of *CP* (*k* = 2), results confirmed that higher levels of CU traits involved a better recognition of fear (Schwenck et al., 2014; Woodworth & Waschbusch, 2007). Also, CU levels did not affect emotional processing (Schwenck et al., 2014), and lower levels of CU traits implied lower recognition of sadness as compared to healthy controls (Schwenck et al., 2014). Moreover, it was the intensity of CP and not the intensity of CU traits what seemed to determine poorer fear recognition (Woodworth & Waschbusch, 2007).

*Other combinations* yielded similar results. In groups with CD/ODD, emotional affect was given by high or low levels of ODD, so that at high levels of CU in both conditions, there was poorer recognition of fear in the ODD− group and worse recognition of anger in ODD+ (Hartmann & Schwenck, 2020). In other cases, it was the presence of several dimensions of psychopathy that was associated with the observed deficits; in this regard, CU traits determined worse recognition of sadness and high GM implied worse recognition of disgust (Levantini et al., 2022a).

Despite the few studies that looked at psychopathic personality as a whole, it seems to be this combination (e.g., CP + PP), and not exclusively CU traits, which seems to show more deficits in emotional recognition within clinical groups. In fact, when looking only at CU traits, it seems that the intensity of disruptive behavioral disorders could make the difference in emotion recognition, rather than the presence of CU traits. Thus, CU traits would be more associated within the (sub)clinical groups with better and faster fear recognition, with effects ranging from moderate (Ezpeleta et al., 2017) to large (Martin-Key et al., 2018).

##### Comorbid ADHD

Co-occurrence rates between CP and ADHD are particularly high (Hudec & Mikami, 2017). That is the reason why data concerning comorbidity of ADHD were reported when available for some clinical groups (*k* = 20). Two studies did not report any test or control analysis for comorbid ADHD (Martin-Key et al., 2020; O’Kearney et al., 2017), and some others did not control for comorbid ADHD (Aghajani et al., 2021; Klapwijk et al., 2016; Levantini et al., 2022a; Muñoz et al., 2021). Yet, in most of the included studies that reported comorbid levels of ADHD, its potential effect was tested or controlled for (Ezpeleta et al., 2017; Hartmann & Schwenck, 2020; Kohls et al., 2020a; Martin-Key et al., 2017, 2018, 2021; Peticlerc et al., 2019; Schwenck et al., 2014; Woodworth & Waschbusch, 2007), as it was the effect of ADHD medication (Bours et al., 2018; Dadds et al., 2018; Schwenck et al., 2011). When ADHD was considered, there were no significant differences in the analyzed variables between children with and without comorbid ADHD, or results were replicated in the same direction when ADHD or hyperactivity was removed or controlled for (Dadds et al., 2011; Fairchild et al., 2009, 2010; Schwenck et al., 2011), even in community samples (Peticlerc et al., 2019). Additional studies also reported that ADHD medication did not influence the observed results (Bours et al., 2018; Dadds et al., 2018; Schwenck et al., 2011).

#### Emotional Stimuli Presented

##### Type of Stimulus Presented (Facial, Vocal or Bodily)

Most of the studies included in this review addressed emotional recognition and/or processing using human facial stimuli (*k* = 46). Of these, only 7 have found no deficits associated with the presence of psychopathic traits, either conceptualized as specific dimensions or as a whole (Aghajani et al., 2021; Kahn et al., 2016; Kohls et al., 2020b; Martin-Key et al., 2020; Milone et al., 2019; O’Kearney et al., 2017; Schwenck et al., 2011). Milone et al. (2019) and Sharp et al. (2014) focused the analysis on a specific facial region, i.e., the eyes, finding mixed results (against and supporting respectively), regarding the impairment of emotional recognition in the presence of psychopathic traits.

Emotional recognition of human bodily postures was also explored (Muñoz, 2009; Martin-Key et al., 2021; Wolf & Muñoz, 2014). Except Martin-Key et al. (2021), which only found an atypical fixation behavior (i.e., arm preference) in high CD males, the remaining studies found deficits in emotion recognition in the presence of psychopathic traits. Deficits were also replicated for vocal stimuli. Deficits in the vocal recognition of sadness were the most common (Gillen et al., 2018; Stevens et al., 2001), but there were also deficits in the recognition of the vocal affect of anger and fear in relation to CU traits (Gillen et al., 2018), and fear in the presence of psychopathic traits (Blair et al., 2005).

De Ridder et al. (2016) deserves special mention for locating the assessment of emotional recognition in a valid ecological environment. Results showed the same accuracy levels for distress emotions between low and high CU groups, but results revealed differences in overestimating intensity of anger and distress emotions in the staff, especially when participants were misbehaving. Finally, it should be noted the use of non-human stimuli in some studies. Emoticons were used in Ezpeleta et al. (2017) and dolls with detachable faces in O’Kearney et al. (2017, 2020). Except for O’Kearney et al. (2017), deficits were found in accuracy and reaction time for mixed (e.g., happy-sad) and simple emotions in clinical ODD groups.

By synthesizing, the relationship between the presence of psychopathic traits and deficits in emotional recognition was found in all types of emotional stimuli studied.

##### Intensity and Duration of Stimuli

Regarding *intensity*, there was no agreement in the results found across the analyzed studies. Hence, in non-clinical groups, some authors found that higher levels of psychopathic traits require a clearer emotional stimulus to be recognized (Blair et al., 2001). However, Bowen et al. (2014) found that the presence of psychopathic traits determined a worse recognition of disgust at medium to high intensities. Furthermore, when considering only CU traits, participants recognized anger rated with very low and very high intensity better.

In the case of clinical groups, the results overall indicated that recognition accuracy was better for high intensity stimuli at both high and low CU levels. However, CD/CU+ groups benefited less from the increased intensity of emotional expressions (Pauli et al., 2021). In some cases, CU and emotional intensity interacted to predict initial eye preference for surprise, which increased with emotional intensity and was larger for high CU participants (Martin-Key et al., 2018).

The effects of *duration* were only analyzed in Lemos Vasconcellos et al. (2014), who found that the duration of the stimuli presentation affected fear recognition in those groups that scored high on psychopathic traits, with moderate effect sizes. Fear recognition was worse in the shortest experimental condition (200 ms) compared to the other conditions. No other differences between groups reached statistical significance; yet effect sizes also demonstrated a moderate difference for fear recognition at 500 ms and 1 s, with worse performance for the High PP group, and small-to-moderate difference for sadness and surprise at 500 ms, with worse performance for the Low PP group.

#### The Role of Attention

The pattern of attention and eye fixation to regions of the face or body as a mediating factor of emotional recognition has been a factor studied in some articles of the present review (*k* = 11). Within the *non-clinical groups*, results tend to indicate that at higher levels of CU traits, an aberrant attentional pattern towards the eye region is observed in the number of fixations (Dadds et al., 2008; Demetriou & Fanti, 2022), and in the duration of eye interactions and on the first attentional focus to the eye region (Dadds et al., 2008), which could determine a deficit in the recognition of fear (Dadds et al., 2008) or for all emotions in general (Demetriou & Fanti, 2022). In addition, high levels of CU traits were related to a greater attention to the mouth area as compared to the eye area (Demetriou & Fanti, 2022), corroborating this dysfunctional attention pattern, which does not seem limited to the eye region. Some longitudinal studies found that low levels of eye contact influenced the development of CU traits 1 year later, but only when maternal sensitivity was low, despite considering that the attentional role was not related to emotional recognition (Bedford et al., 2017).

Within *(sub)clinical groups*, Dadds et al. (2011) were the first to address the hypothesis of attentional biases linked to emotion recognition. Specifically, they analyzed this relationship across gender, finding that males with high CU traits showed consistent impairments in eye contact towards their parents. In addition, high CU fathers but not mothers showed less eye contact with their child. Furthermore, higher levels of eye contact in child-parent and parent–child dyads were related with increased recognition of fear. Confirming previous results regarding fear, Bours et al. (2018) found that higher scores on psychopathic traits within the ODD/CD group were related to shorter time to first eye fixation for fear recognition. Considering also disruptive behavioral disorders, Hartmann and Schwenck (2020) noted that higher levels of CU traits predicted the number of mistakes in fear trials in which only the eyes were presented but only when CP were low. In contrast, the effects of CU traits varied according to CD status and sex, where males with lower levels of CU traits showed the most atypical fixation behavior (Martin-Key et al., 2021). In the opposite direction, Muñoz et al. (2021) found that children high on CU traits did not show a significant deficit in reflexive gaze to the eye region for fearful faces. In fact, children high on CU traits performed more poorly in labelling fearful faces accurately, only when the mouth region was primed.

Taking other emotions into account, Billeci et al. (2019) concluded that in children with a DBD diagnosis, high levels of CU traits were associated with lower average length of fixations on the eye areas of sad faces, which in turn predicts lower levels of sadness recognition. Hartmann and Schwenck (2020) linked high CU traits with more mistakes in the sad trials when only the mouth of the stimuli was presented, whilst Levantini et al. (2022a) found a smaller number of fixations to the eyes for sad faces. Indeed, Levantini et al. (2022a) were the ones who used a wider variety of emotions considering the influence of overall psychopathic traits. Regarding gaze pattern, they found that CU traits were negatively associated with length or first fixation to the mouth of angry faces and eyes of disgusted faces. INS was positively associated with the number of fixations and average length of each fixation to the eyes of angry faces, whilst GM was negatively associated with the number of fixations to the eyes of angry faces and positively associated with the number of fixations to the mouth of angry faces. Studying surprise, CU traits in the CD group were related to a reduced attention to the eyes for surprise faces. Also, CU traits interacted with emotional intensity so that the greater the emotional intensity, the greater the tendency for initial eye fixation on the surprise faces. Notwithstanding the aforementioned results, revealing substantial differences in terms of attentional fixation pattern, some authors concluded that the attention was no related with emotion recognition (Bedford et al., 2017; Hartmann & Schwenck, 2020; Martin-Key et al., 2021).

In sum, it seems that attention show aberrant patterns in children/adolescents with psychopathic traits, involving different patterns regarding the areas of interest analyzed (i.e., eyes or mouth) with moderate effect sizes for both AOI. The presence of clinical disorders and the region examined (eyes or mouth) also seemed to moderate the results.

#### Moderator Variables

Various studies have investigated the influence of certain moderating variables in the relationship between psychopathic traits and emotional recognition. The most researched have been age, sex, intelligence quotient (IQ), ethnic origin and the presence of anxiety and maltreatment.

##### Age

Several articles considered the effect of age in the analyses conducted (Blair & Coles, 2000 [11–14; *M* = 12.4]; Hartman & Schwenck, 2020 [8–14; *M* = 10.4]; Martin-Key et al., 2018 [13–18], 2021 [13–18]; Rehder et al., 2017 [6–7]; White et al., 2016 [3–7; *M* = 4.82]; Wolf & Muñoz, 2014 [11–16; *M* = 14.3]; Woodworth & Waschbusch, 2007 [7–12.78; *M* = 9.81]). Significant effects of age as a moderator variable were found in two studies. In one of them, the authors found that older age implied greater emotional recognition for static and dynamic facial stimuli (Kimonis et al., 2016 [3–7; *M* = 4.82]). In other modalities such as vocal, the results indicated that older age was associated with better fear recognition even within the high psychopathic group who showed worse recognition patterns than the comparison group (Blair et al., 2005 [11.8–15.5]). In addition, the importance of assessing emotional recognition at an early age has been noted, since deficits in emotional recognition may predict an increase in the later development of CU traits (Bedford et al., 2017).

Furthermore, by taking a global perspective, there is a noticeable tendency for no discernible differences in emotion recognition between clinical groups with high and low levels of psychopathic traits, or even with psychopathic traits dimensionally measured as age increases. Thus, whilst all studies but one (O’Kearney et al., 2017 [4–8]) conducted in samples of both preschool and elementary-school children (i.e., up to 12 years old), did find significant differences in emotion recognition, when we consider adolescent samples (i.e., from 12 years old), several studies reported no significant differences (Aghajani et al., 2021 [15–19]; De Ridder et al., 2016 [*M* = 14.8]; Kahn et al., 2016 [14–18; *M* = 17.03], 2017 [3–7; *M* = 4.82]; Klapwijk et al., 2016 [12–20; *M* = 15.5]; Martin-Key et al., 2017 [13–18], 2020 [13–18], 2021 [13–18]), a finding that may be moderated by increasing age.

##### Sex

Regarding sex, there is an expected over- representation for the male perspective, probably due to the higher prevalence of psychopathic traits in this population. Yet, one study showed that, within the clinical groups, there were no differences across sex in CU traits (Kohls et al., 2020a). Given this inequality in considering sex, the need to pay attention to gender differences became evident.

Sex was controlled for in some studies (Blair & Coles, 2000; Ezpeleta et al., 2017; Hartmann & Schwenck, 2020; Kimonis et al., 2016; White et al., 2016; Woodworth & Waschbusch, 2007). Some studies observed a trend suggesting that belonging to the female sex entails a higher level of emotional recognition (Kohls et al., 2020a; Pauli et al., 2021; Rehder et al., 2017). Nevertheless, in those studies that specifically examined sex differences, the results were in the same direction as male studies. Considering only female samples, all samples analyzed were clinical and included morphed or dynamic facial stimuli, showing again mixed results. Whereas in Martin-Key et al. (2020) there were no significant correlations between CU traits in the CD group and emotion recognition, in the other two studies, the presence and intensity of psychopathic and CU were associated with specific deficits or advantages in emotion recognition. As for recognition accuracy, participants with CD and high on psychopathic traits showed impaired recognition of sadness (Fairchild et al., 2010) while samples of girls with CP/CU+ recognized fearful expressions better and mistook sad faces as disgusted faces (Schwenck et al., 2014). In contrast, participants with low CU traits recognized less sad expressions and identified fearful faces as disgusted faces (Schwenck et al., 2014). As for reaction time, girls with CP/CU+ did not differ from CP/CU− and comparison group (CG), but girls with CP/CU− reacted more slowly than CG to faces developing happy, sad and fearful expressions (Schwenck et al., 2014).

In terms of attention, sex played an important role in determining eye contact, where mothers of children with CU traits did not show impairments but fathers did, with less eye contact with their children (Dadds et al., 2011). Regarding atypical fixation, CD males with lower levels of CU showed the most atypical fixation behavior compared with CD girls (Martin-Key et al., 2021). According to eye preference, with exception of disgust, females showed greater total eye preferences as compared to the mouth than male for all emotions (Martin-Key et al., 2018).

##### Socioeconomic Status (SES)

The socioeconomic status (SES) has been taken into account in several of the analyzed articles. In most cases, it was assessed using measures related to parental income, educational level, and occupational status. However, it is important to note that SES varied according to the different countries included in each of the studies. Most of the articles categorize SES into ranges: low, medium, and high.

SES has been utilized as a descriptive variable of the sample (e.g., Rehder et al., 2017), employed for creating homogeneous groups based on sociodemographic variables (e.g., Fairchild et al., 2010), and sometimes included in the analyses as a control variable (e.g., Bedford et al., 2017).

No evidence was found for SES as a moderating variable in the association between psychopathic traits and emotion recognition. Nevertheless, it is worth emphasizing that except for two articles, which found no differences regarding SES (Bowen et al., 2014; Martin-Key et al., 2020), most studies showed a clear relationship between clinical group membership and lower SES as compared to typically developing groups (Dadds et al., 2018; Fairchild et al., 2009, 2010; Martin-Key et al., 2017, 2018, 2021). Furthermore, for CU individuals within clinical samples, SES levels were the lowest among all reported groups (Aghajani et al., 2021; Pauli et al., 2021; Woodworth & Waschbusch, 2007), a result that was replicated in forensic and at-risk samples (De Ridder et al., 2016; Kimonis et al., 2016).

##### IQ

The role of IQ was controlled for in some studies (Aghajani et al., 2021; Blair et al., 2001; Kahn et al., 2017; Woodworth & Waschbusch, 2007). Only one study mentioned the role of IQ as a potential moderator of recognition. Both in the general sample and in a subsample of psychopathic individuals, Blair et al. (2005) found a directly proportional relation between IQ and impaired emotional recognition. In both cases, IQ was presented as a facilitator of the recognition of the vocal affects of fear and disgust.

##### Ethnic Origin

Only the study conducted by Rehder et al. (2017) examined whether differences in emotional recognition might be due to ethnicity, with large effect sizes for this variable. They compared a group of European American (EA) children with a group of African-American (AA) children. The presence of CU traits did not influence the differences in performance between the CP and the CP + CU group. For specific emotion recognition, only EA children within the CG showed better recognition for happy faces compared to children within the clinical groups. Also, ethnicity was associated with SES, moderating the differences between CG, CP and CP + CU groups. Only CG of EA children with no extreme poverty showed better accuracy for overall emotion recognition and better recognition for happy faces.

##### Anxiety and Maltreatment

Anxiety has been an internalizing factor studied in moderating the relationships between the presence of psychopathic traits (especially CU traits) and emotional recognition (*k* = 2). Kahn et al. (2017) found deficits by analyzing specific emotions, with large effect sizes. Results indicated better fear recognition at lower levels of anxiety and a deficit in the recognition of disgust in the presence of high levels of anxiety.

Anxiety linked to maltreatment in clinical groups was studied in Dadds et al. (2018), who found that the relationship between the presence of CU traits and emotional recognition was different according to the previous history of maltreatment and levels of anxiety. Thus, only in those cases with zero or negligible history of maltreatment and lower levels of anxiety, CU traits were associated with worse emotional recognition. This poorer emotional recognition was not limited to fear or sadness, and negative correlations were found for all the analyzed emotions except happiness.

Thus, despite limited studies, it appears that levels of maltreatment do not seem to correlate with emotional recognition, whilst levels of anxiety seem to play an important role. In fact, there seems to be a difference depending on whether we consider clinical or non-clinical groups. While in clinical groups low levels of anxiety determine worse emotional recognition (Dadds et al., 2018), in non-clinical groups, it was the presence of higher levels of anxiety what influenced worse emotional recognition (Kahn et al., 2016).

## Discussion

The aim of the present systematic review was to assess emotion recognition in children and adolescents with varying levels of psychopathic traits. To this end, we considered some significant variables that could act as relevant moderators (e.g., socio-demographics, sample type, presence of problematic behaviors and clinical disorders, stimuli type and conditions of presentation). We also included studies measuring eye fixation behavior during the emotional tasks to investigate whether atypical attentional patterns had a significant role in emotion recognition. It was overall intended to provide compelling evidence on the topic, accounting for variability in results, but also for robust patterns that can be delineated from the current findings, and which will be detailed below.

### Pervasive or Specific Deficits Across Psychopathic Traits

Findings pointed out that variability is the main relevant result attained. Yet, we found a recurrent trend for pervasive deficits in emotion recognition considering both overall psychopathic traits and specific CU, particularly in non-clinical samples. This result would be in line with past metanalytical results (Dawel et al., 2012), and would provide additional support for a general impairment in emotion recognition (e.g., Dadds et al., 2018). However, in line with preliminary Blair’s hypothesis (1995, 2006), specific deficits in recognizing distress emotions (i.e., particularly fear and also sadness) were also found, a result that also emerges in a previous meta-analysis conducted in adult and young populations (Wilson et al., 2011), and which was repeatedly observed in most of the studies included in the present review. These deficits affected both accuracy and processing, measured as the reaction time, and were more notable for complex than simple emotions (e.g., Bowen et al., 2014; Sharp et al., 2014). Importantly, the observed deficits emerged across all modalities of emotion presented (facial, vocal, bodily, non-human) and all emotional stimuli used, a result that has been recently replicated in a study specifically aimed at examining the role of stimulus characteristics in emotion recognition related to CU traits (Powell et al., 2023).

Notwithstanding these findings, some patterns of mixed results were also found. Hence, some studies showed no relationship between emotion recognition and CU traits (e.g., Aghajani et al., 2021; Hartmann & Schwenck, 2020; Martin-Key et al., 2017, 2020), whilst others found an increased ability for emotion recognition in relation to CU traits (e.g., Bowen et al., 2014), a result that might be dependent of the assessment measurement used (Hartmann & Schwenck, 2020).

Accounting for CU subdimensions, or even CU variants, could help to clarify these results. However, in the present review only two studies considered the subdimensions of CU traits (Kimonis et al., 2016; Moore et al., 2019), with no conclusive results probably due to the lack of homogeneity in clustering the subdimensions of CU traits. It could be suggested that emotion recognition deficits are more related with both callousness and uncaring dimensions, whilst unemotional traits would show a distinctive pattern of results, with improved emotion recognition for disgust and surprise (Moore et al., 2019). These results would be in line with previous studies raising the usefulness of the callousness-uncaring combination in the CU conceptualization (Cardinale & Marsh, 2020), with some suggesting reconsiderations to the inclusion of unemotional traits as a facet of CU traits (Hawes et al., 2014). However, much more research is needed to clarify the role of each subdimension, if some suppression effect might be expected when using a total score, and whether measurement biases could be influencing these results. More research is also needed regarding the CU variants, with only one study in this review specifically focused on CU variants (Bennett & Kerig, 2014), and two more addressing the combination between CU traits and anxiety (Kahn et al., 2017) or maltreatment (Dadds et al., 2018) as a proxy of this CU distinction. Results again are mixed, probably due to methodological variability, as observed in a previous review on the topic (Craig et al., 2021). Thereby, some results suggest that primarily CU individuals, characterized by low levels of anxiety, could show an enhanced emotion recognition for some specific emotions like anger (Bennett & Kerig, 2014) or fear (Kahn et al., 2016), whilst secondary CU would be related with improved recognition of disgust (Bennett & Kerig, 2014). Others, in contrast, suggest that at low levels of anxiety (i.e., primary CU) CU traits would be related with pervasive emotion recognition deficits, affecting all emotions except happiness (Dadds et al., 2018). Considering the important distinction between primary and secondary variants, and the role that anxiety may play as moderator in emotion recognition, interesting questions arise about the precise nature of emotional processing among youths within each variant. As suggested by Craig et al. (2021) it is possible, for example, that differences may be apparent in processing emotional information about others, but not necessarily about the self. Additional research, aimed at clarifying differences in emotional processing and recognition between the two variants is particularly encourage. It might help to elucidate how anxiety, and other early adversity variables, may influence in the development of emotion recognition and awareness.

Notably, the pattern of mixed results observed in the current review was more frequently observed for CU traits, but not when other psychopathy dimensions were examined, with one exception (i.e., Gillen et al., 2018). One potential explanation of these results, beyond potential measurement biases, could be the presence of different forms of disruptive behavior, including CP, ODD and CD. Results showed that it was the combination between disruptive behavior and multidimensional psychopathic traits which was related to a dampened ability for emotion recognition (Fairchild et al., 2009, 2010; Levantini et al., 2022a), with some specific deficits for CU traits, related with worse recognition of sadness, and for GM, related with worse recognition of disgust (Levantini et al., 2022a). Further, within this (sub)clinical samples, some studies revealed that it might be the level of disruptive behavior instead of CU traits which mostly determined the impaired emotion recognition (Woodworth & Waschbusch, 2007). In this regard, several studies showed no relationship with CU traits in different (sub)clinical groups (Aghajani et al., 2021; Khols, Baumann et al., 2020; Ezpeleta et al., 2017; Kohls et al., 2020b; Klapwijk et al., 2016; Martin-Key et al., 2017, 2020, 2021; Milone et al., 2019; Schwenck et al., 2011). Less consistent but also relevant was the presence of an enhanced emotion recognition in high CU participants within (sub)clinical samples, particularly in the recognition of fear (e.g., Ezpeleta et al., 2017; Schwenck et al., 2014; Woodworth & Waschbusch, 2007). These results were also replicated for reaction time, with less time needed to recognize emotions in general (Klapwijk et al., 2016) and fear in particular (Martin-Key et al., 2018).

These results raise two important questions to be clarified in future research. First, the importance of testing the potential moderator role of disruptive behavior, particularly in relation to CU traits. The extracted results suggest that CU traits are indeed associated with deficits in emotion recognition, with a more pronounced effect in the absence or at low levels of disruptive behavior (Woodworth & Waschbusch, 2007). Second, the need to account for all psychopathy dimensions. Certainly, CU traits has been the most analyzed dimension, with great advances on delineating a potential developmental precursors of psychopathy (Frick, 2022; Frick et al., 2014). However, some voices have claimed that all psychopathy dimensions should be better conceptualized within the construct, which may help to better understand how psychopathic traits identify a distinctive group of problematic children and adolescents (Salekin, 2017, 2022). It implies that developmental models of psychopathic personality, and their related research, also include additional psychopathy dimensions to clearly disentangle how they develop, and which mechanistic processes might be underlying (i.e., similar or distinctive than those traditionally observed for CU traits).

Even though within this review a lower number of studies focused on psychopathic personality as a multidimensional construct, the results extracted were more robust, clearly suggesting a close relationship with emotion recognition deficits (e.g., Blair & Coles, 2000; Blair et al., 2001; Bowen et al., 2014; Gillen et al., 2018). This result was even more remarkable within problematic or (sub)clinical samples (e.g., Fairchild et al., 2009, 2010; Levantini et al., 2022a). It should be noted that most of these studies relied on a global psychopathy score, which limits the possibility of examining potential distinctive results across dimensions. Even in some studies assessing all psychopathic traits, there was a specific focus on CU traits (e.g., Dadds et al., 2006, 2008, 2011) with GM and INS dimensions being used as potential covariates. When different dimensions were specifically examined within the psychopathy construct, CU traits seem to be more related with impairments in emotion recognition. GM traits, on the other side, would be to some extent also related with better accuracy—and higher fixation to the mouth—for angry faces, when controlling for the other two dimensions (Gillen et al., 2018; Levantini et al., 2022a). As observed in previous studies, it seems that the combination between all psychopathy dimensions is what better identifies a more serious group of problematic children and youths (e.g., Bergstrøm & Farrington, 2022; Burke et al., 2022; Colins et al., 2022; Fanti et al., 2018; López-Romero et al., 2021, 2022). According to the current results, it could be the case that these individuals, with overall high levels on psychopathic traits, would also be the most impaired in terms of emotion recognition. Interestingly, these results held in both correlational and between-groups designs. In this regard, even though correlational designs allow accounting for all the dimensional spectrum (e.g., Kahn et al., 2016; Sharp et al., 2014), when high versus low psychopathic groups are considered, deficits in emotion recognition became more evident (e.g., Blair et al., 2005; Fairchild et al., 2010).

As was previously mentioned, the noticeable variability in the assessment of psychopathic traits could have impacted the aforementioned mixed results. For instance, there were variation across studies in terms of the version/informant (i.e., parent-, teacher-, and self-reports), the dimension used to measure psychopathic traits (e.g., Total score versus CU), the dimensional versus categorical conceptualization of the psychopathy dimension or the cut-offs used to classify individuals based on the psychopathy score. Interestingly, results seem to suggest that mixed results might not be directly related with the instrument. Instead, variation in results could be to some extent related with the informant, a result that clearly emerged with the ICU, with emotion recognition deficits being more evident with parent- and teacher-reported version of the ICU. It could be the case that response biases (e.g., social desirability) could affect more the self-report of personality aspects that entails negative connotations, or that a lack of insight or even the unwillingness to respond (e.g., tendency to lie) could have an impact on self-reports (Ray et al., 2013). This hypothesis that was not well-supported on previous research (Kelley et al., 2018; Miller et al., 2011), not either in the current review, with other self-reported measures, assessing different psychopathy dimensions (i.e., APSD, YPI) showing significant results in relation to emotion recognition. Similarly, in a recent meta-analytic review, results showed a greater relationship between psychopathic traits and negative outcomes (e.g., externalizing problems) for other- than self-reported psychopathy, although differences were not to a substantial extent (Mendez et al., 2023). The dimension addressed with the instrument (e.g., CU versus the multidimensional construct, with firmer results for the later), and other moderator variables (e.g., sex, ethnicity, anxiety, sample type) seem to also affect the results more than the instrument itself. Additional studies specifically aimed at comparing different measures would be of great interest. Future research involving all psychopathy dimensions across different samples and settings should be particularly encouraged, as well as additional comparisons between dimensional versus categorical approaches, considering similar cut-off points. Clarifying the best way to conceptualize and assess psychopathic traits will help to further examine those underlying mechanisms that, as in the case of emotion recognition, may affect socioemotional functioning, having an impact on later behavioral, emotional and psychosocial adjustment.

### Attention Biases in Emotion Recognition

When categorizing facial emotions, attention is typically directed towards critical facial features, most notably the eyes and the mouth (Eisenbarth & Alpers, 2011; Wells et al., 2016). In fact, a failure to attend to these regions may lead to difficulties in judging the expressed emotions. It has been suggested that one mechanism whereby amygdala dysfunction, largely linked to psychopathic personality (Yang & Raine, 2018), may contribute to pervasive emotion recognition deficits is the abnormal attention patters to the face regions, particularly the eyes (Dawel et al., 2012), a result that has been replicated across laboratory tasks and real-life interactions.

The results of the current review support Dadds’s theory of pervasive deficit in emotion recognition (Dadds et al., 2006, 2008), which would be derived by a dysfunction on attentional mechanisms that underlie emotion recognition. Psychopathic traits were associated with atypical eye scan paths for emotional faces in children and adolescents (Billeci et al., 2019; Dadds et al., 2008; Martin-Key et al., 2018) and with different patterns of attention regarding an alternative area of interest; i.e., the mouth (Demetriou & Fanti, 2022; Hartmann & Schwenck, 2020). These results also corroborate previous findings observed in adult samples (e.g., Dargis et al., 2018; Gillespie et al., 2015). The small number of articles in the current review dealing with traits other than CU (only Levantini et al., 2022a account for each psychopathy dimension) makes it difficult to establish a robust connection with different psychopathy dimensions in samples of children and adolescents. Moreover, less is known about the role of attention in clinical or forensic samples, but preliminary results seem to go in the same direction, i.e., the presence of clinical disorders could be moderating the associations (Hartmann & Schwenck, 2020). The same occurs when the area of interest is considered. Hence, in clinical and forensic groups, atypical fixation patterns are observed not only to the priority face areas of interest, i.e., eyes (Billeci et al., 2019) and mouth (Levantini et al., 2022a), but also deficits were seen according to CD status when the area of interest was bodily posture (Martin-Key et al., 2021).

### The Role of Moderator Variables

The identification of emotional expressions can be affected by several factors that have been researched and considered in the field of emotion recognition. The most widely studied have been age, sex and ethnic origin. The results, in line with what has been discussed throughout the study, have been mixed.

According to the biopsychosocial perspective, the development of various aspects of emotional and behavioral functioning occurs through interactions and coactions at multiple levels of analysis, including genetic aspects, neural and cognitive activity, behavior and social and cultural environments (Gottlieb, 2007). In line with this last point, patterns of socialization or upbringing may influence children's emotional competencies through verbal and non-verbal information communicated by parents to children (Dunbar et al., 2015). In an attempt to clarify the matter, different ethnic stimuli have been used as emotional tasks and ethnicity has been considered in the sample description of the studies; yet, the role of ethnicity has been barely considered when interpreting the results due to a lack of studies specifically accounting for this variable. Recently, Laukka and Elfenbein (2021) pointed out evidence of advantages related to belonging to the same ethnic group, with vocal emotion being more accurately recognized when expressers and perceivers were from the same cultural group. These differences in terms of ethnicity were preliminary supported in this review, with one study showing that the observed results were moderated by ethnicity, with European-American participants showing better accuracy in emotion recognition when CP were not present (Rehder et al., 2017).

As for age, the trend towards greater emotional recognition with increasing age has become apparent in recent years in samples of children (Schaan et al., 2019) and adults (Hayes et al., 2020). These results held in the current review for groups of children and adolescents with psychopathic traits in several modalities of presentation such as vocal (Blair et al., 2005) and image (Kimonis et al., 2016). However, it seems that magnitude and direction of age effects may be influenced by elements of task design in emotional recognition tasks, with larger age effects in combined tasks (i.e., static and dynamic; Hayes et al., 2020; Kimonis et al., 2016), an interaction that should be considered. Of note, most of the studies conducted in childhood covered school-aged children, so caution is needed when extending these findings to preschool samples.

Undoubtedly, the sex variable has been the most considered and the one that has generated most debate about its implication in emotional recognition. Many studies underline the advantage of woman in decoding emotions (Olderbak et al., 2018) and better outperformance of females in clinical (Kohls et al., 2019) and normative youth samples (Bek et al., 2022; Wells et al., 2016) and adult samples (Abbruzzese et al., 2019). Further, some investigations about sex differences were conducted to understand the neural basis of emotional processing and recognition (Derntl et al., 2010; Li et al., 2020). However, in the present review, the results obtained in terms of sex, especially when analyzing studies focused on female samples, show the same trends and deficits as their male counterparts (Fairchild et al., 2010; Martin-Key et al., 2020; Schwenck et al., 2014). Similar results were obtained in mixed samples that controlled for sex (Blair & Coles, 2000; Ezpeleta et al., 2017; White et al., 2016; Woodworth & Waschbusch, 2007). Additional research conducted by sex, or specifically in female samples, would be extremely needed to corroborate whether similar deficits are present, or an alternative pattern of dysfunction might be identified. These studies should be in line with future research aimed at examining the structure of psychopathic personality in girls, clarifying whether the current conceptualization of psychopathy, largely based on males’ representation, accurately replicates for different female samples (Haneveld et al., 2022).

In sum, while is crucial to consider the moderating variables mentioned above, it is equally important not to overlook the influence of task characteristics. The interaction among all these factors seems to provide a better explanation of the differences observed in the results (Pauli et al., 2021; Martin-Key et al., 2018; Wells et al., 2016). This fact would be in line with the premise that there exist multiple contributors to emotional recognition, including genetic, biochemical and environmental factors (Christov-Moore et al., 2014). Accounting for most of them, including other relevant moderators such as IQ family SES, could help to clarify the marked pattern of mixed results.

### Strengths, Limitations and Recommendations for Future Research

This is the first review that examines the entire literature up to 2022 on the relation of emotion recognition and psychopathic traits in childhood and adolescence. The obtained results reflect relatively strong trends in the data that at least deserve to be the subject of future research. Some of the most robust conclusions indicate the presence of deficits in emotional recognition—especially distress emotions—and raise the need to explore the role of attention and sociodemographic variables as moderators of emotional recognition, as well as to consider all psychopathy dimensions and the role of disruptive behavioral disorders in understanding emotion recognition in relation to psychopathic traits.

Nevertheless, the results attained in this systematic review and the conclusions that have been reached should be assessed in the light of some important limitations. The most remarkable concerns the high heterogeneity found in the different studies. It is hard to identify a unique source for this variability, but there are some possible factors that might be of influence. Multiple emotions were presented, but not the same across all studies. Most of them considered the basic emotions (Ekman, 1992), but at times, the emotions chosen were in response to the researcher's objectives (e.g., distress emotions, complex emotions). Intensity at presentation, complexity and exposure times were variable too, both being characteristics relevant for emotion recognition. Also, occasionally, emotional stimuli were presented several times, so the learning effect cannot be dismissed. The environmental conditions (e.g., light, noise, spaces) of the experimental setup and the way information is collected (e.g., self-registration, third part collection) can lead to differences in the results. The wide range of different instruments used to assess psychopathic traits, with different informants (i.e., teachers, parents, self-report), the consideration of psychopathic traits from a dimensional versus a categorical perspective, or the arbitrary cut-off point used to determine the presence/absence of psychopathic traits, may also lead to inconsistencies in the results. As a result of this variability, at times, the shortage of studies made it challenging to extract robust results and draw generalizable conclusions. In addition, some studies did not provide enough information for replication. In this regard, while certain recognition deficits were replicated across stimuli and presentation modalities, which is relevant to generalization, future research would benefit from homogenizing experimental conditions and promoting replication studies across diverse samples, contexts, and settings.

Other factors that affect the variability in results include the limited sample size in some of the included studies and the non-differentiation of the sample with respect to sex. A better understanding of this phenomenon inevitably requires the establishment of differentiated samples between boys and girls and the recruitment of a sufficient sample size to be able to draw reliable conclusions, particularly when between-groups designs are considered. Also important is to continue examining the longitudinal association between emotion recognition and psychopathic traits, as most of the studies included in this review were cross-sectional and hindered the possibility to interpret the directionality of the effects. New longitudinal research will help to disentangle the potential causal mechanisms, addressing linked deficits in other relevant brain structures and functional areas. Hence, complementary measures of emotional processing, including psychophysiological recordings (i.e., heart rate, skin conductance), are particularly needed to provide further evidence on underlying mechanisms that might be influencing the relationship between psychopathic traits and emotion recognition. Future research would also benefit from including preschool samples, as the available results are scarce in this developmental period. This would help to elucidate whether deficits in emotion recognition in early childhood could be somehow explained by the presence of psychopathic traits beyond age-related reasons (i.e., increased ability to recognize different emotions as children grow up). Finally, additional suggestions for future research would include stimuli with different emotional intensities. Limiting the recognition methodology only to images of high emotional intensity may not provide a clear picture of emotional recognition difficulties, whilst contemplation of various emotional intensities may be best suited to reveal sensitivity and more subtle differences in recognition (Adolphs & Tranel, 2004). More attention should be also paid to other modalities such as vocal affect, as they represent a great complement to facial and bodily stimuli in socialization processes. Lastly, promotion of cross-national and cross-cultural studies with the aim of outlining cultural differences in emotional recognition should be also encouraged.

Some other limitations concerning this study should also be outlined. It covers a wide period of time (i.e., more than 20 years), and two distinctive developmental periods (i.e., childhood and adolescence), which results in an appreciable number of studies, with multiple results to be extracted. This fact, along with the inclusion of mixed samples, makes complex to provide a finer-grained extraction, with studies specifically examined by age, sex or sample type. Relatedly, to address all the intended objectives, an extensive search equation was used, providing a great number of results to be screened. The great number of eligible studies required an organized extraction. For the current study, and based on our objectives, results were organized based on the psychopathy dimension analyzed, and the assessment of attention deficits, but other forms or organization could be possible, including the sample type, or the developmental period. However, because some studies included mixed samples, these alternative forms of organization were finally discarded. The inclusion of adolescent samples led to include some participants older than 18, resulting in a wide age range. Yet, their inclusion was justifiable as they were part of well-defined adolescent samples (e.g., high school, samples within juvenile forensic systems that include participants up to 21 years old). Finally, restricting the search to published studies (i.e., excluding grey literature) may raise the likelihood of publication biases due to the file drawer effect. The inclusion of multiple results, in different directions, could have attenuated this effect but the potential influence of this kind of biases cannot be diminished when interpreting the results.

### Theoretical and Practical Implications

Emotion recognition of facial expressions represents a crucial component of human social interaction, allowing the observer to infer another's emotional state and adjust their behavior (Blair, 2003b). This review provides important clinical, social and scientific implications. On a clinical and scientific level, it raises the need to attend to this problem at an early age, as we already know that psychopathic traits can be reliable identified early in development (Colins et al., 2014; López-Romero et al., 2019) and, what is even more important, that these traits could be more malleable at childhood when behavioral patterns are not firmly established. Disentangling the mechanisms underlying the development of psychopathic traits will provide additional insight about a construct that has proved its value for child and youth CP. This, in turn, will help to improve the development of more tailored preventive and intervention approaches, aimed at restraining high-risk patterns of behavioral maladjustment, with related benefits in the policy and social fields. However, more research is needed to clarify how preventive interventions could be improved from research in emotion recognition, particularly in early childhood.

In (sub)clinical samples, parent training seems to be the option that works best to reduce conduct problems (Romero et al., 2023). Recently, attempts have been made to improve behavioral parent training interventions to increase efficacy for children with CP + CU (Dadds et al., 2019; Kimonis et al., 2019; Waschbusch et al., 2019) with some promising results for high CU children evidenced in a recent meta-analysis (Perlstein et al., 2023). Based on previous research on the association between parenting practices and psychopathic traits (e.g., Waller et al., 2018), these tailored programs emphasized the importance of improving the quality of parent–child relationships by increasing sensitive and receptive parenting interactions (Kimonis et al., 2019). Aligning with prior recommendations of translating emotion-related evidence to prevention (e.g., Izard, 2002), some recent interventions have also incorporated an element of distress cue and emotion recognition training, with the goal of increasing socioemotional skills (e.g., empathy) in children with high CU traits. Interestingly, promising results have been reported in this regard (Fleming et al., 2022), even when considering attention deficits as the target of the intervention (Muñoz et al., 2021). Because previous research has suggested that parenting practices could be moderating the association between psychopathic traits and emotion recognition deficits (Levantini et al., 2022b), additional knowledge on these interactions would serve to continue refining parenting programs with socioemotional components specifically tailored to children and adolescents with psychopathic traits.

Yet, these promising implications at the practical level should also be interpreted with caution. Hence, some studies included in this review showed no deficits (e.g., Martin-Key et al., 2020), or even better recognition (e.g., Ezpeleta et al., 2017) in relation to psychopathic traits. This is particularly important within the (sub)clinical groups, with psychopathic traits, and not just CU, being linked with impairments in emotion recognition. However, high CU individuals tend to be the target of the current interventions intended to reduce child CP (Perlstein et al., 2023), whilst other psychopathic dimensions have been overlooked in intervention. Also, if psychopathic individuals show enhanced abilities for emotion recognition, as revealed but some studies in this review (e.g., Ezpeleta et al., 2017; Klapwijk et al., 2016; Martin-Key et al., 2018), this kind of interventions may come with some iatrogenic results. Improving the current knowledge on emotion recognition in relation to psychopathic personality, accounting for all its dimensions and potential moderators, would be decisive to keep moving forward at the practical level, with refinements in evidence-based programs that really account for deficits in this population.

Homogenizing research conditions and favoring replication studies could help in this purpose, allowing the establishment of more robust and clearer conclusions on the association between emotion recognition and psychopathic traits and, in turn, on the transfer of those results to the applied context.

## Conclusions

This is the first systematic review specifically focused on the association between psychopathic traits, accounting for all its dimensions, and emotion recognition deficits in children and adolescents. Results overall showed impairments in emotion recognition in relation to psychopathic traits. These results revealed pervasive deficits across emotions, although they were more marked for distress emotions, including fear or sadness, with deficits being replicated across all modalities of emotion presentation and all stimuli used. Importantly, when disruptive behavior is present, overall psychopathic traits, beyond CU, seem to account for emotion recognition deficits. Attentional patterns seemed biased in children and adolescents high on psychopathic traits and involve different patterns regarding the areas of interest analyzed, including the eyes or the mouth. Notwithstanding these results, the present review is characterized by a great heterogeneity in study designs and task conditions, hampering the establishment of firm conclusions. Considering the importance of this research for developmental models of psychopathic traits, replication studies, based on more standardized study characteristics, are particularly encouraged.

### Supplementary Information

Below is the link to the electronic supplementary material.

Supplementary file1 (DOCX 104 KB)

## Data Availability

The materials that support the findings of this study are available from the corresponding author, LLR, upon request.

## Footnotes

1 When no other clarification is made, the terms “children” or “childhood” overall refer to both early (i.e., preschool) and middle childhood (i.e., elementary school), including children up to age 12.

2 These dimensions, particularly the interpersonal and behavioral, have been differently labelled in previous literature as it is the case of “grandiose-deceitful” for the interpersonal (Andershed, et al., 2002; Colins et al., 2014) or “daring-impulsive” (Salekin & Hare, 2016), “impulsive-irresponsible” (Andershed et al., 2002) for the behavioral dimensions. To be consistent across the study, we have assumed GM, CU and INS acronyms, as defined in the main text, to refer to interpersonal, affective and behavioral psychopathic traits respectively.
