# Supplementary material for: Emotion Recognition Deficits in Children and Adolescents with Psychopathic Traits: A Systematic Review
Source: Clin Child Fam Psychol Rev. 2024 Jan 19;27(1):165–219. doi: 10.1007/s10567-023-00466-z (PMC10920463; doi:10.1007/s10567-023-00466-z)
Supplement: Supplementary file 1 — Supplementary file1 (DOCX 104 KB) [file 10567_2023_466_MOESM1_ESM.docx]

**Emotion Recognition Deficits in Children and Adolescents with Psychopathic Traits:**

**A Systematic Review**

**Supplementary Material**

**Table S1** PRISMA 2020 Reporting Checklist

**Table S2** *Detailed Search Strategies*

**Table S3** *Main Study Characteristics*

**Table S4** *Studies´s Quality Assessment by Using the Appraisal Tool for Cross-sectional Studies (AXIS)*

**Table S5** *Studies´s Quality Assessment by Using the Critical Appraisal Skills Program (CASP) for Longitudinal Studies*

**Table S6** *Excluded Items after Abstract and Full Text Review and Reasons (k=24)*

Beatriz Díaz-Vázquez^a^, Laura López-Romero*^a^, and Estrella Romero^a^

^a^ Department of Clinical Psychology and Psychobiology, Universidade de Santiago de Compostela, Spain

***Correspondence concerning this article** should be addressed to L. López-Romero, Department of Clinical Psychology and Psychobiology. Facultade de Psicoloxía. Campus Vida, Universidade de Santiago de Compostela (Spain). Phone: (+34) 881 813735. E-mail: [laura.lopez.romero@usc.es](mailto:laura.lopez.romero@usc.es)

**Table S1**

PRISMA 2020 Reporting Checklist

| Section/topic | # | Checklist item | Reported on page # |
| --- | --- | --- | --- |
| TITLE | | |  |
| Title | 1 | Identify the report as a systematic review, meta-analysis, or both. | 1 |
| ABSTRACT | | |  |
| Structured summary | 2 | Provide a structured summary including, as applicable: background; objectives; data sources; study eligibility criteria, participants, and interventions; study appraisal and synthesis methods; results; limitations; conclusions and implications of key findings; systematic review registration number. | 2 |
| INTRODUCTION | | |  |
| Rationale | 3 | Describe the rationale for the review in the context of what is already known. | 3 - 7 |
| Objectives | 4 | Provide an explicit statement of questions being addressed with reference to participants, interventions, comparisons, outcomes, and study design (PICOS). | 7 - 9 |
| METHODS | | |  |
| Eligibility criteria | 5 | Specify the inclusion and exclusion criteria for the review and how studies were grouped for the syntheses. | 10 - 12 |
| Information sources | 6 | Specify all databases, registers, websites, organizations, reference lists and other sources searched or consulted to identify studies. Specify the date when each source was last searched or consulted. | 9 - 10 |
| Search strategy | 7 | Present the full search strategies for all databases, registers and websites, including any filters and limits used. | Table 1 & Table S2 |
| Selection process | 8 | Specify the methods used to decide whether a study met the inclusion criteria of the review, including how many reviewers screened each record and each report retrieved, whether they worked independently, and if applicable, details of automation tools used in the process.. | 12 - 13 |
| Data collection process | 9 | Specify the methods used to collect data from reports, including how many reviewers collected data from each report, whether they worked independently, any processes for obtaining or confirming data from study investigators, and if applicable, details of automation tools used in the process. | 12 - 13 |
| Data items | 10a | List and define all outcomes for which data were sought. Specify whether all results that were compatible with each outcome domain in each study were sought (e.g. for all measures, time points, analyses), and if not, the methods used to decide which results to collect. | 13 - 14 |

| Section/topic | # | Checklist item | Reported on page # |
| --- | --- | --- | --- |
|  | 10b | List and define all other variables for which data were sought (e.g. participant and intervention characteristics, funding sources). Describe any assumptions made about any missing or unclear information. | 13 - 14 |
| Study risk of bias assessment | 11 | Specify the methods used to assess risk of bias in the included studies, including details of the tool(s) used, how many reviewers assessed each study and whether they worked independently, and if applicable, details of automation tools used in the process. | 13 |
| Effect measures | 12 | Specify for each outcome the effect measure(s) (e.g. risk ratio, mean difference) used in the synthesis or presentation of results. | 13 - 14 |
| Synthesis methods | 13 | Describe the processes used to decide which studies were eligible for each synthesis (e.g. tabulating the study intervention characteristics and comparing against the planned groups for each synthesis (item #5). | 13 - 14 |
| Reporting bias assessment | 14 | Describe any methods used to assess risk of bias due to missing results in a synthesis (arising from reporting biases). | - |
| Certainty assessment | 15 | Describe any methods used to assess certainty (or confidence) in the body of evidence for an outcome. | - |
| RESULTS | | |  |
| Study selection | 16a | Give numbers of studies screened, assessed for eligibility, and included in the review, with reasons for exclusions at each stage, ideally with a flow diagram. | 14 & Figure 1 |
|  | 16b | Cite studies that might appear to meet the inclusion criteria, but which were excluded, and explain why they were excluded. | Table S6 |
| Study characteristics | 17 | For each study, present characteristics for which data were extracted (e.g., study size, PICOS, follow-up period) and provide the citations. | 14 – 17 & Table S3 |
| Risk of bias within studies | 18 | Present data on risk of bias of each study and, if available, any outcome level assessment (see item 12). | 17 - 18 -Table S4 & Table S5 |
| Results of individual studies | 19 | For all outcomes, present, for each study: (a) summary statistics for each group (where appropriate) and (b) an effect estimate and its precision (e.g. confidence/credible interval), ideally using structured tables or plots. | 18 - 34 |
| Results of syntheses | 20a | For each synthesis, briefly summarize the characteristics and risk of bias among contributing studies. | 18 - 34 |
|  | 20c | Present results of all investigations of possible causes of heterogeneity among study results. | 18 - 34 |
| Reporting biases | 21 | Present assessments of risk of bias due to missing results (arising from reporting biases) for each synthesis assessed. | - |
| Certainty of evidence | 22 | Present assessments of certainty (or confidence) in the body of evidence for each outcome assessed. | - |
| Section/topic | # | Checklist item | Reported on page # |
| DISCUSSION |  | . |  |
|  | 23a | Provide a general interpretation of the results in the context of other evidence | 34 - 42 |
|  | 23b | Discuss any limitations of the evidence included in the review. | 42 - 45 |
| Discussion | 23c | Discuss any limitations of the review processes used. | 44 - 45 |
|  | 23d | Discuss implications of the results for practice, policy, and future research. | 44 - 46 |
| OTHER INFORMATION | | |  |
|  | 24a | Provide registration information for the review, including register name and registration number, or state that the review was not registered | 10 |
| Registration and protocol | 24b | Indicate where the review protocol can be accessed, or state that a protocol was not prepared. | 10 |
|  | 24c | Describe and explain any amendments to information provided at registration or in the protocol. | Prospero |
| Support | 25 | Describe sources of financial or non-financial support for the review, and the role of the funders or sponsors in the review. | Title page |
| Competing interests | 26 | Declare any competing interests of review authors. | Title page |
| Availability of data, code and other materials | 27 | Report which of the following are publicly available and where they can be found: template data collection forms; data extracted from included studies; data used for all analyses; analytic code; any other materials used in the review. | Upon request |

**Table S2**

*Detailed Search Strategies*

| **Database** | **Full Boolean search equation** | **Additional filters** |
| --- | --- | --- |
| PSYCINFO | ALL((callous* unemotion*) OR (CU) OR (psychopathy) OR (psychopathic)) AND ALL((emotion* recognition) OR (emotion* process*) OR (emotion* identification) OR (eye gaze) OR (eye track*) OR (eye fix*) OR (facial emotion*) OR (emotion* attent*)) AND ALL((child*) OR (adolesc*)) AND la.exact("Spanish" OR "English") AND age.exact("Neonatal (birth-1 Mo)" OR "Infancy (2-23 Mo)" OR "Preschool Age (2-5 Yrs)" OR "School Age (6-12 Yrs)" OR "Adolescence (13-17 Yrs)" OR "Childhood (birth-12 Yrs)") AND po.exact("male" OR "inpatient" OR "outpatient" OR "human" OR "female") AND (su.exact("Adolescence (13-17 yrs)" OR "Childhood (birth-12 yrs)" OR "School Age (6-12 yrs)" OR "Preschool Age (2-5 yrs)" OR "Infancy (2-23 mo)") AND PEER(yes)) AND PEER(yes) | - |
| SCOPUS | TITLE-ABS-KEY (("callous* unemotion*" OR cu OR psychopathy OR psychopathic) AND ("emotion* recognition" OR "emotion* process*" OR "emotion* identification" OR "eye gaze" OR "eye track*" OR "eye fix*" OR "facial emotion*" OR "emotion* attent*") AND (child* OR adolesc*)) AND (LIMIT-TO (LANGUAGE, "English")) | - |
| PUBMED | ((callous* unemotion* OR CU OR psychopathy OR psychopathic) AND (emotion* recognition OR emotion* process* OR emotion* identification OR eye gaze OR eye fix* OR facial emotion* OR emotion* attent*) AND child* OR adolesc*)) | Language: English/ Spanish  Species: Humans  Age: birth – 18 years |
| WOS | (callous* unemotion* or CU or psychopathy or psychopathic) (All Fields) and (emotion* recognition or emotion* process* or emotion* identification or eye gaze or eye track* or eye fix* or facial  emotion* or emotion* attent*) (All Fields) and (child* or adolesc*) (All Fields) | Languages: English or Spanish |

**Table S3**

*Main Study Characteristics*

| **Study** | **Main purpose** | **Design** | **Sample definition** | **% males** | **Location (Ethnicity)** | **Psychopathy dimension** | **Emotion Recognition** | | | | **Attention biases** | |
| --- | --- | --- | --- | --- | --- | --- | --- | --- | --- | --- | --- | --- |
|  |  |  |  |  |  |  | **Stimuli** | **Exposure duration** | **Nº of blocks/trials** | **Response format** | **Yes/No** | **Measure/**  **Device** |
| Aghajani et al. (2021) | 1. Examine neural processing of recognizing and resonating socioemotional content among CD offenders with LPE relative to CD offenders without LPE and healthy controls | Cross-sectional  Between-groups | Juvenile offenders and healthy controls | 100% | The Netherlands, Europe  (NR) | CU  (LPE proxy) | Six facial expressions of fear and sadness of ethnically diverse young men  (*RaFD*) | 2.47s | 12/6 | Infer the emotional state | No | - |
| Bedford et al. (2017) | 1.Test (1) whether infants’ mother-directed gaze, maternal sensitivity (T1), and childhood ER (T3) predict later CU (T2, T4)  2. Examine whether ER mediates the association of infant gaze and maternal sensitivity with later CU | Longitudinal  Correlational | Full-term infants and their families from the Durham Child Health and Development Study (DCHDS) | ~ 49% | UK  (57% African American; 43% European American) | CU | Photographs of elementary-aged children posing various facial expressions  (*ACES*) | NR | 1/8  (2 x emotion) | Verbal response to the question “Does he/she feel happy, sad, mad, scared or no feeling?” | Yes | Infants’ mother-directed gaze (FFSFP) |
| Bennet & Kerig (2014) | 1. Investigate ER, emotion dysregulation and emotional numbing among youth characterized by primary and acquired CU | Cross-sectional  Between-groups | Youths recruited from a juvenile detention center | 73.38% | USA  (57.6% white/Caucasian; 4.3% Black/African American; 24% Hispanic/Latino, 3.1% Native American/Alaska; 5% Pacific Islander/Native Hawaiian; 1.2% Asian/American; 4.3% multiracial; | CU | Four photographs for each analyzed emotion from  (*The Emotion Recognition Task*) | 3s | 1/20  (4 x emotion) | Identify the emotion (verbal) | No | - |
| Billeci et al. (2018) | 1. Examine the role of CU traits in determining emotional processing in children with a DBD diagnosis | Cross-sectional  Correlational | A clinical group of children with an ODD/CD disorder and a CG of children with no current or past diagnosis or psychiatric disorder | 100% | Italy, Europe  (NR) | CU | 24 images (4 actors, 6 emotions)  (*NimStim Set of Facial Expressions)* | 4s | NR | Identify the emotion (screen) | Yes | Eye Tracker SMI RED 500 |
| Blair & Coles (2000) | 1. Explore the relationship between the ability to recognize facial expressions and observed affective impairment and behavioral problems | Cross-sectional  Correlational  Between-groups | Participants attending a mainstream school | 56.36% | UK  (55% Caucasian; 20% Afro-Caribbean; 21.82% Asian) | GM/CU, INS | Photographs of all 6 facial emotions  (*The Expression Recognition Hexagon Stimuli*) | 3s | 6/30 | Identify the emotion (verbal) | No | - |
| Blair et al. (2001) | 1. Investigate whether children with psychopathic tendencies (PP) show a similar insensitivity for fearful expressions than observed in adult populations, and whether this insensitivity also extends to sad expressions | Cross-sectional  Between-groups | Participants recruited from three schools for boys with emotional and behavioral difficulties, and comprehensive schools in a high unemployment area. | 100% | NR  (NR) | GM/CU, INS | Three photographic-quality continua for each emotion. A neutral face gradually morphed through 20 5% increment stages into one of the six prototypical expressions  (*The Emotion Expression Multimorph Task*) | 3s | 1/18  (6 x emotion) | Identify the emotion (verbal) as soon as it is recognized | No | - |
| Blair et al. (2005) | 1. Investigate whether boys with psychopathic tendencies show impairment in the processing of sad and fearful vocal intonations | Cross-sectional  Correlational  Between-groups | Boys recruited from three schools for boys with emotional and behavioral difficulties | 100% | NR  (97.7% Caucasian; 2.3% Asian, all in CG) | GM, CU, INS | 6 bisyllabic concrete nouns with neutral meaning (e.g., carpet), emotionally spoken by one male and one female voices  (*Vocal Affect Recognition Test*) | NR | 1/120 | Identify the emotion | No | - |
| Bours et al. (2018) | 1. Examine emotional face recognition in a direct comparison of male adolescents with ASD, or ODD/CD, and a CG of typically developing individuals.  2. Analyze the role of psychopathic traits in emotional face recognition | Cross-sectional  Between-groups | Participants recruited from clinical institutions with a diagnosis of ASD or ODD/CD. Participants from the CG recruited from community | 100% | The Netherlands  (NR) | GM, CU, INS | Static images of emotional and neutral faces  (*NimStim Set of Facial Expressions)* | 6s | 2/30 | Identify the emotion (screen) | Yes | Eye-Tracker Tobii 1750 |
| Bowen et al. (2014) | 1. Examine recognition of facial affect across all emotions and intensities  2. The role of psychopathic traits, CD and offense severity in explaining variation in ER performance | Cross-sectional  Between-groups | Young offenders who had shown delinquent behavior that had brought them in contact with the criminal justice system, and control adolescents from local comprehensive schools and youth centers | 100% | UK  (NR) | GM, CU, INS | A series of 150 slides displaying facial expressions, with 6 targets (3 males, 3 females). The six emotional expressions were morphed to their matching neutral expression (0% emotion) to display faces at 25%, 50%, 75%, and 100% emotional intensity  (*Facial Emotion Recognition Task*) | NR | 1/150 | Select the number on the screen that corresponds with the identified emotion | No | - |
| Dadds et al. (2006) | 1. Test the relationship of fear recognition and eye gaze to psychopathic traits in children and adolescents | Cross-sectional  Correlational  Between-groups | Children attending schools in Sydney | 100% | Sydney, Australia  (NR) | CU  GM-INS (AB) | Four adult faces representing the six emotions  (*UNSW* *Facial Emotion Task)* | 2s | 3/24 (free-gaze, eye-gaze, mouth-gaze; 4 x emotion) | Participants recorded the emotion portrayed from a list of six emotions | No | - |
| Dadds et al. (2008) | 1. Test the findings from the previous Dadds et al. (2006) study, by having participants view and categorize emotional faces using eye-tracker equipment to measure gaze behaviors under free-, eye- and mouth-directed conditions | Cross-sectional  Between-groups | Boys attending a private school | 100% | Sydney, Australia  (European, Asian) | CU  GM-INS (AB) | Six faces (two adult, two adolescents, two child) presented in Power Point slides  (*UNSW* *Facial Emotion Task)* | 2s | 1/36 (free gaze; 6 x emotion)  2/12 (eye-gaze, mouth-gaze; 2 x emotion) | ER Condition: Choose the emotion from a list presented in participants’ booklets  Eye-gaze condition: identify the emotion aloud | Yes | Tobii 1750 binocular Eye Tracker |
| Dadds et al. (2011) | 1. Examine whether the impaired eye contact in a characteristic of CP children with AB and CU traits in real life settings | Cross-sectional  Correlational  Between-groups | Referrals to UNSW child mental health centers | 100% | Sydney, Australia  (NR) | CU  GM-INS (AB) | Emotional faces presented on a computer monitor  (*UNSW* *Facial Emotion Task)* | 1s | NC | Identify the emotion | Yes | Child-mother, child-father, mother-child, father-child eye contact dyads |
| Dadds et al. (2018) | 1. Test whether maltreatment history and anxiety levels moderated the relationship between levels of CU traits and ER skills | Cross-sectional  Correlational | Referrals to the Child Behavior Research Clinical at the UNSW and Royal Far West child health center | 72% | Sydney, Australia  (64.6% Caucasian) | CU | Six faces (two adult, two adolescents, two child) presented on a computer monitor  (*UNSW* *Facial Emotion Task)* | 500ms | 1/60 | Identify the emotion from a list of 5 emotions presented on the screen (with keyword or verbally) | No | - |
| De Ridder et al. (2016) | 1. Assess institutionalized adolescents’ empathic accuracy in their ability to infer the emotions experienced by familiar adults in their natural interactions.  2. Investigate whether high CU adolescents would globally underestimate distress, and not anger, intensities.  3. Explore how empathic accuracy is related to adolescents’ own behavior, and own affective and relational experience | Intensive longitudinal (EMA)  Correlational | Institutionalized adolescents recruited as part of a larger study conducted in youth welfare and juvenile justice institutions | 82% | Switzerland, Europe  (55% Swiss) | CU | Four items assessing anger (two items) and distress (two items) perceived in staff members. Participants reported it 4 times per day (morning, noon, afternoon, evening), during 8 days  (*Ecological Momentary Assessment*) | - | 8/4 | Infer the degree of anger and distress  (Answers reported in a PDA) | No | - |
| Demetriou & Fanti (2022) | 1. Investigate eye-gaze behavior of children with varying levels of CU traits.  2. Analyze their ability to accurately identify emotional expressions | Cross-sectional  Between-groups | Children who scored reliably high and low on CU traits across informants, selected from a large sample (n = 1283) preschool and primary school children | 54.24% | Cyprus, Europe  (NR) | CU | 32 static images of four adults and four children (50%) depicting four emotional expressions (*MPAFC*) | 3s | 1/32 | Identify the emotion in a choice screen with accuracy ratings | Yes | Tobii X120 eye-tracking |
| Ezpeleta et al. (2017) | 1. Identify difficulties in recognizing and attending to emotions in children with different levels of ODD and CU traits | Cross-sectional  Between-groups | Children participating in the sixth follow-up of a large-scale longitudinal study of behavior problems | 51.6% | Spain, Europe  (93.4% Non-Hispanic white; 3.1% Hispanic-American; 3.4% Other) | CU | Emoticons expressing emotions (angry, sad, happy and fearful) and non-emotions (neutral) presented in a computer-based go/no go task | 1.5s | 1/120  (24 x emotion) | Press the key on the keyboard each time an emoticon with an emotion was shown, and to inhibit the response to press when a neutral emoticon was presented | No | - |
| Fairchild et al. (2009) | 1. Compare adolescents with early-onset CD (EO-CD) and adolescence-onset forms of CD (AO-CD), and matched healthy controls (CG) in terms of facial expression recognition accuracy.  2. Analyze the role of psychopathic traits in emotion recognition | Cross-sectional  Between-groups | Participants recruited from secondary schools, pupil referral units and the Cambridge Youth Offending Service | 100% | UK, Europe  (82.1%-97.6% White) | GM, CU, INS | Six facial expressions morphed across continua spanning six expression pairs: happiness-surprise, surprise-fear, fear-sadness, sadness-disgust, disgust-anger, anger-happiness  (*The Emotion Hexagon Task*) | 5s | 6/30 | Select one of the six expression labels displayed on the keyboard | No | - |
| Fairchild et al. (2010) | 1. Determine whether deficits in emotion recognition are present in girls with CD.  2. Analyze the role of psychopathic traits in emotion recognition | Cross-sectional  Between-groups | Participants recruited from secondary schools, pupil referral units and the Cambridge Youth Offending Service | - | UK, Europe  (92%-96.67% Caucasian) | GM, CU, INS | Six facial expressions morphed across continua spanning six expression pairs: happiness-surprise, surprise-fear, fear-sadness, sadness-disgust, disgust-anger, anger-happiness  (*The Emotion Hexagon Task*) | 5s | 5/20 | Label the facial expression | No | - |
| Gillen et al. (2018) | 1. Determine whether psychopathic traits are differentially related to face and voice processing.  2. Examine whether psychopathic traits are related to emotional intelligence, cognitive and affective empathy.  3. Examine whether emotional skills may affect the psychopathy-recidivism relation | Cross-sectional  Correlational | Participants placed in a regional detention facility | 66% | NR  (55% Black; 45% White) | GM, CU, INS | Facial subset: Photographs of people displaying facial expressions of one of the four emotions.  Vocal tone subset: Audio clip of a person stating a standardized sentence with a voice tone exhibiting one of the four emotions  (DANVA-II) | 5s | NR | Determine, in a time-frame allotted, the emotion being displayed | No | - |
| Hartman & Schwenck (2020) | 1. Assess whether CU traits are associated with deficits in emotion recognition independent of externalizing behavior  2. Examine whether such deficits can be explained by aberrant attention | Cross-sectional  Correlational | Participants recruited from outpatients clinics, through advertisements in local newspaper, mainstream schools, sports clubs and via mail-shots | 60.64% | Germany, Europe | CU | Paradigm 1:  Three photographs of the same face, depicting once sad, fearful, and angry facial expressions. Stimuli consisted of nine female and nine male Caucasian models  (*RaFD*)  Paradigm 2:  Block 1: Single facial (5 male, 5 female) expressions.  Block 2: Single facial (5 male, 5 female) expressions with the upper (eye-condition) or the lower (mouth-condition) halve of the face being visible | Paradigm 1: -  Paradigm 2: 2s | Paradigm 1:  3/36  Paradigm 2:  1/30  1/60 | Paradigm 1: Recognize a target emotion among three emotional expressions (press one of three buttons). They receive auditory feedback for wrong answers and could only proceed once they chose the correct stimulus  Paradigm 2: Identify the emotion by pressing one of three buttons, as fast as possible | Yes | Remote eye-tracker RED 250 |
| Khan et al. (2016) | 1. Examine the association between Emotional Intelligence (EI) and CU traits in a sample of incarcerated adolescents | Cross-sectional  Correlational | Participants recruited from a southwestern US juvenile maximum-security detention facility | 83.7% | Southwestern USA (74.5% Hispanic or Latino; 9.2% American Indian or Alaska Native; 6.4% White; 5.7% Black or African-American; 4.2% Mixed) | GM, CU, INS | 32 pictures (8 faces) from the perceived emotion branch of a Experiential Emotional Intelligence  (*MSCEIT-YV-R*) | NR | 1/32 | Rate facial expressions to determine, in a scale of 1-5, how the person feels on five distinctive emotions | No | - |
| Khan et al. (2017) | 1. Test whether anxiety moderated the association of CU traits with self-report and computerized measures of affective (emotional reactivity) and cognitive (affective facial recognition and ToM) empathy | Cross-sectional  Correlational | Participants recruited from three secure detention facilities | 100% | Southeastern USA  (79% African American, 14% Caucasian, 5% Hispanic, 2% Other) | CU | 36 facial expressions from the NimStim displayed by 6 adult faces varying in ethnic composition and gender  (*UNSW* *Facial Emotion Task)* | 2s | 1/32 | Identify the emotion from a list of 6 emotions presented on the screen | No | - |
| Kimonis et al. (2016) | 1. Test the factor structure, reliability and validity of parent- and teacher-report scores on the preschool version of the ICU.  2. Test the association between ICU scores and emotion processing | Cross-sectional  Correlational | Children recruited from both mainstream and high-risk preschools | 52% | Cyprus, Europe  (NR) | CU | Static condition:  40 prototypical (100%) facial expressions of both men and women from the Pictures of Facial Affect Series expressing the analyzed emotions  (*The Ekman Emotional Expression Task*)  Dynamic condition:  40 dynamic prototypical facial expressions morphed from neutral to one of the five expressions | 1s | Static : 1/40 (10 x emotion)  Dynamic: 1/40 (8 x emotion) | Label each emotion aloud. The researchers recorded the child’s response on a keyboard using designated keys | No | - |
| Klapwijk et al. (2016) | 1. Compare the neural correlates of cognitive and affective empathy between youth with ASD and youth with CD/CU+ | Cross-sectional  Between-groups | Participants with ASD recruited from specialized child psychiatric centers.  Participants with CD/CU+ recruited from a juvenile detention center and a forensic psychiatry unit.  CG recruited through local advertisement | 100% | The Netherlands, Europe  (NR) | CU | Angry and fearful faces  (RaFD) | 2.47s | 36/6 | Infer the emotional state/judge their own emotional response, using a three-button response device | No | - |
| Kohls, Baumann et al. (2020) | 1. Characterize emotion processing skills in a large sample of girls and boys with CD compared to typically developing controls (CG) | Cross-sectional  Between-groups | Participants from the European “Neurobiology and Treatment of Female Conduct Dirsorder” (FemNAT-CD) project, recruited through community outreach (mainstream school), mental health clinics, welfare institutions and youth offending services across 10 countries in Europe | 36.42% | Europe  (NR) | CU | Five facial expressions morphed across continua spanning six expression pairs  (*The Emotion Hexagon Task*) | 3s | 5/30 | Identify the emotion (mouse-click) | No | - |
| Kohls, Fairchild et al. (2020) | 1. Explore neurocognitive diversity of emotion functioning in CD through a clinically motivated, person-centered bottom-up analytic approach | Cross-sectional  Between groups | Participants from the European “Neurobiology and Treatment of Female Conduct Dirsorder” (FemNAT-CD) project, recruited through community outreach (mainstream school), mental health clinics, welfare institutions and youth offending services across 10 countries in Europe | 36.42% | Europe  (NR) | CU | Five facial expressions morphed across continua spanning six expression pairs  (*The Emotion Hexagon Task*) | 3s | 5/30 | Identify the emotion (mouse-click) | No | - |
| Lemos Vasconcellos et al. (2014) | 1. Compare adolescents with and without psychopathic traits in terms of their ability to recognize emotional facial expressions, at different time periods for the stimuli | Cross-sectional  Between-groups | Adolescents at a correctional institution whose records indicated different levels of severe and frequent antisocial behavior based on a psychologist interview | 100% | Brazil  (NR) | GM, CU, INS | 24 photographs of professional actors representing the multiracialism of Brazilian population  (*FERBT)* | 200ms  500ms  1s | NR/24 | Verbally indicate the emotion | No | - |
| Levantini et al. (2022) | 1. Explore the association between CU traits and emotion processing while controlling for the other psychopathy dimensions.  2. Explore whether narcissism and impulsivity were associated with emotion processing impairments after controlling for the other dimensions | Cross-sectional  Correlational | Participants from a specialized service for children with behavioral problems | 100% | Italy, Europe  (NR) | GM, CU, INS | Passive viewing task with 24 trials.  Two males and two females represented six facial emotions  (*NimStim Set of Facial Expressions)* | 4s | 1/24 (4 x emotion) | Select the emotion that best described the displayed expression | Yes | Eye Tracker SMI RED 500 |
| Lui et al. (2016) | 1. Address whether CU traits in adolescents are associated with deficits in cognitive and/or affective empathy, and how abilities such as affective perspective tacking, and facial emotion recognition may be associated with those deficits | Cross-sectional  Correlational | Participants enrolled in a residential program for adolescents who have dropped out high schools due to behavioral, academic, economic and personal/family difficulties | 67.96% | Southeast USA  (57.3% White; 33% Black; 9.7% other) | CU | 36 child, teen and adult faces depicting the analyzed emotions  (*UNSW* *Facial Emotion Task)* | 2s | 1/216 | Identify the expressed emotion from a list of six response choices | No | - |
| Martin-Key et al. (2017) | 1. Compare adolescents with CD and typically developing controls (CG) in different measures of empathy accuracy, accounting for different levels of CU traits within the CD group | Cross-sectional  Between-groups | Participants recruited through Youth Offending Services and pupil referral via poster advertisements and referrals from case workers, and by sending out information packs to students at mainstream schools and colleagues | 100% | UK, Europe  (87% Caucasian) | CU | 12 test clips (2 instances per emotion) with actors filmed talking about autobiographical experiences in which they had felt discrete primary emotions | *M* 144s  (61-158s) | 1/24  (2 x emotion) | Name the predominant emotion displayed in the video clip from a list of six primary emotions. | No | - |
| Martin-Key et al. (2018) | 1. Investigate facial emotion recognition in male and female adolescents with CD and varying levels of CU traits, and typically developing youths (CG).  2. Investigate whether CU traits are associated with impaired emotion recognition performance and attention to the eyes  3. Examine whether atypical fixation patterns mediate fear recognition deficits in those high on CU traits | Cross-sectional  Between-groups | Participants recruited through Youth Offending Services and pupil referrals units, via referrals with case workers and through mainstream schools and colleges via mail-shots. | 51.49% | UK, Europe  (91.09% Caucasian) | CU | Dynamic:  56 stimuli (8 models, 4 males) expressing the emotions depicted from neutral to full emotion transition  Static: 100 stimuli (4 models, 2 males) depicting emotional facial expressions at four emotion intensities (30%, 50%, 70%, 100%) | 1000ms | 4/39 | Select the emotion that best describe the displayed emotion (mouse click) | Yes | Eye Link 1000 eye tracker |
| Martin-Key et al. (2020) | 1. Investigate whether females with CD show empathy deficits using a ecologically-valid task.  2. Examine whether CU traits influenced empathic accuracy within the CD group.  3. Test for sex differences, using data from Martin-Key et al. (2017) | Cross-sectional  Between-groups | Participants recruited through Youth Offending Services and pupil referral units via referrals from caseworkers, and through mainstream schools and colleges via mail-shots | - | UK, Europe  (92.3% Caucasian) | CU | 12 test clips (2 instances per emotion) with actors filmed talking about autobiographical experiences in which they had felt discrete primary emotions | *M* 144s  (61-158s) | 1/24  (2 x emotion) | Name the predominant emotion displayed in the video clip from a list of six primary emotions. | No | - |
| Martin-Key et al. (2021) | 1. Examine the recognition of static and dynamic emotional body postures  2. Investigate whether individuals with CD show atypical fixation behavior when processing body postures, and whether fixation behavior mediates deficits in emotional recognition  3. Examine whether CU traits are associated with impaired body posture recognition and atypical fixation behavior | Cross-sectional  Between-groups | Participants recruited through Youth Offending Services and pupil referrals units, and through mainstream schools and colleges via mail-shots. | 50% | UK, Europe  (91.67%) | CU | 42 video clips with two actors (one male, one female) representing emotions. Actors’ faces were blurred to eliminate emotional information portrayed via facial expressions. Static stimuli were derived from the dynamic sequences by extracting one highly identifiable frame | 3000ms | 2/42 | Select the label that best described the emotion (mouse click) | Yes | EyeLink 1000 eye tracker |
| Milone et al. (2019) | 1. Explore the relationships among CU traits, cognitive and emotional dimensions of empathy, emotion recognition (basic, social and complex emotions), and history of maltreatment.  2. Examine the difference between CD patients with low and high CU traits of the different components of empathy | Cross-sectional  Correlational  Between-groups | Patients consecutively referred to the Department of Child and Adolescent Psychiatry of Scientific Institute “Stella Maris” | 100% | Italy, Europe  (NR) | CU | 28 photographs of the eye region of the face  (CET) | NR | 1/10 | Choose which of four words best describe what the person in the picture is feeling | No | - |
| Moore et al. (2019) | 1. Replicate previously observed correlations between CU traits and emotion recognition.  2. Examine the genetic and environmental correlation between CU traits and emotion recognition.  3. Determine the proportion of any significant phenotypic correlation that are due to the shared genetic and/or environmental etiology | Cross-sectional  Correlational | Participants drawn from two epidemiological twin samples | 46% | Mid Atlantic Region, USA  (100% Caucasian) | CU | Morphed pictures displaying each target emotion in 10 different gradients from 0% to 100% of the target emotion  (FELT) | 500 ms | 1/360 | Indicate the depicted emotion out of the six possible emotion options | No | - |
| Muñoz (2009) | 1.Determine whether children with CU traits evince the same deficits in identifying fear body postures as they do with fear faces | Cross-sectional  Correlational | Youths from a disadvantage neighborhood of England | 100% | UK, Europe  (NR) | CU | 1.24 adult faces  2.18 body postures of two men and two women with blurred face | NR | 1/24 faces  1/24 body | Label the expressed emotion from a list of six response choices (for both conditions) | No | - |
| Muñoz et al. (2021) | 1.Investigate whether children with CU traits show less reflexive attentional orienting towards the eye region of fearful facial expressions | Cross-sectional  Correlational | Youths from four special schools “Emotional and Behavioral Difficulty Schools” | 84% | North East of England, UK, Europe  (96% White British) | CU | 40 individual faces (20 male 20 female), for each emotional expression  (*NimStim Set of Facial Expressions)* | 150 – 3000 ms | 1/55  1/55  1/50  (3 experim. sessions) | Report which facial expression they had been presented | Yes | EyeLink 1000 eye tracker |
| O`Kearney et al. (2017) | 1.Examine the performance of young children across emotional competencies.  2.Examine whether relative deficits in emotional competencies are marked or specific for ODD children with high levels of CU | Cross-sectional  Between-groups | Child recruited with difficult to manage behaviors and from schools as part of a study about how to enhance children`s abilities to better manage difficult emotion | 57% | NR | CU | Dolls with detachable faces that depict happy, sad, angry and afraid expressions in a random order | NR | NR | Point the correct face and label the emotion signaled | No | - |
| O`Kearney et al. (2020) | 1.Examine the role of level of affect arousal/dysregulation in moderating the nature of the relationship between CU and emotional abilities in children with ODD | Cross-sectional  Correlational | Child recruited with difficult to manage behaviors and from schools as part of a study about how to enhance children`s abilities to better manage difficult emotion | 58% | NR | CU | Dolls with detachable faces that depict happy, sad, angry and afraid expressions in a random order | NR | NR | Point the correct face and label the emotion signaled | No | - |
| Pauli et al. (2021) | 1.Check if general emotion recognition performance is poorest in youths with CU/CU+  2.Test the hypothesis that youths with CD/CU+ have specific difficulties with fear and sadness recognition, over and above any general difficulties | Cross-sectional  Between-groups | Participants from the European “Neurobiology and Treatment of Female Conduct Dirsorder” (FemNAT-CD) project, recruited through community outreach (mainstream school), mental health clinics, welfare institutions and youth offending services across 10 countries in Europe | 42% | Europe  (NR) | CU | Five facial expressions morphed across continua spanning six expression pairs  (*The Emotion Hexagon Task*) | 5s | 5/30 | Identify the emotion (mouse-click) | No | - |
| Peticlerc et al. (2019) | 1. Analyze whether CU traits are related to poorer processing of facial expressions of fear and sadness.  2. Examine the specificity of possible genetic relationships, controlling for shared variance between CU and other behavioral problems, as well as the recognition of other emotions | Longitudinal  Correlational | Pair of twins from an ongoing longitudinal study: The Quebec Newborn Twin Study | NR | Canada, North America  (84% European descent) | CU | Visual subtest of the Diagnostic Analysis of Nonverbal Accuracy Scale 2 (*DANVA-II*). 16 adult and 16 child faces showing emotions | 2s | 1/29 | Identify the emotion pressing a button with an emoticon | No | - |
| Rehder et al. (2017) | 1.Examine facial emotion recognition among children characterized by high and low levels of CP and CU behaviours  2. Examine the role of socio-demographic variables in emotional recognition abilities | Longitudinal  Between-groups | Birth cohort of children living in areas of high rural poverty from The Family Life Project | 49.1% | North Carolina and Pennsylvania, USA  (42.5% African American (AA); 57% European American (EA);0.5% other) | CU | The Increasingly Clear Emotions task (*ICE*). 20 adult (half female) facial expressions shifted from neutral to prototypical emotional expressions | NR | 4/20 | Identify the emotion via a forced-choice response format | No | - |
| Schwenck et al. (2011) | 1. Compare cognitive and emotional empathy in different age groups of children with ASD, CD with high or low CU traits (CU+ vs CU-) and a matched CG | Cross-sectional  Between-groups | Children recruited from the Department of Child and Adolescent Psychiatry (University of Würzburg), and local psychiatrist. CG recruited by an advertisement in the local paper | 100% | Germany, Europe  (NR) | CU | 60 film clips with a neutral facial expression changing to an emotional facial expression | 9s | 2/60  (6 x emotion) | Press key and name the emotion | No | - |
| Schwenck et al. (2014) | 1. Compare recognition of morphed emotional faces in girls with CP, with elevated or low CU traits, and matched HC | Cross-sectional  Between-groups | Girls with CP recruited from the Department of Child and Adolescent Psychiatry (University of Würzburg), and local psychiatrist. CG recruited by an advertisement in the local paper | - | Germany, Europe  (NR) | CU | 60 film clips with a neutral facial expression changing to an emotional facial expression | 9s | 2/60  (6 x emotion) | Press key and name the emotion | No | - |
| Sharp et al. (2014) | 1. Test whether CU traits are related to deficits in emotion recognition over and above other psychopathy dimensions.  2. Examine whether this relationship is driven by a specific deficit in recognizing complex rather than basic emotions | Cross-sectional  Correlational | Participants attending 17 elementary schools in the Netherlands | 53.6% | The Netherlands, Europe  (75% Caucasian; 25% other) | GM, CU, INS | 28 photographs of the eye region of adult faces  (CET) | NR | 1/28 | Select the emotion | No | - |
| Stevens et al. (2001) | 1. Investigate the ability of children with emotional and behavioral difficulties to recognize emotional facial expressions and vocal tones | Cross-sectional  Between-groups | Participants attending a school for children with emotional and behavioral difficulties | 100% | NR  (NR) | GM/CU, INS | Facial expressions: 24 photographs of children and adults showing facial expressions  Auditory stimuli: 24 children’s and adults’ voices repeating a sentence with intonations reflecting the emotions | Facial expression: 2s  Auditory stimuli: 4s | 1/24 (facial)  1/24 (auditory) | Identify the emotion | No | - |
| Sylvers et al. (2011) | 1.Examine whether psychopathic traits, specifically CU traits, are associated with preattentive fear- recognition deficits  2.Test the association between CU traits and preattentive recognition deficits in specific to fear  3.Analize if each of the individual psychopathy factors show an incremental contribution above and beyond the contributions of the other factors | Cross-sectional  Correlational | Participants recruited through flyers that targeted preadolescent children who were often in trouble at home and school | 100% | Georgia, USA  (45.5% Caucasian, 44.3% African American, 6.8% Asian 3.4% Hispanic) | GM, CU, INS | The modified (mCFS) task. On a split screen with four quadrants, two different stimuli are presented simultaneously to each eye. A geometric pattern in one and a gradually presented (2% contrast every 100ms) emotional face image in the other, that appears in one of the possible quadrants in the stimulus square | 1s | 1/100  (25 x emotion) | Press a button corresponding to the quadrant where face was presented | No | - |
| White et al. (2016) | 1.Examine emotional expression recognition deficits in very young children with developmentally defined patterns of CU traits | Cross-sectional  Between-groups | Participants drawn from Phase II of the Multidimensional Assessment of Preschoolers (MAPS) Study, which recruited preschoolers from five Chicago-based pediatric clinic waiting rooms. Children were oversampled for disruptive behavior and mothers’ intimate partner violence exposure | 45.1% | Chicago, USA  (45.1% African-American; 31.5% European-American; 22% Hispanic; 1.5% Other) | CU  (LC/PI) | Pictures of adult faces presented on arrays of nine faces (eight neutral and one emotional) | Until response | 3/27 | Identify the emotional face (touch screen) | No | - |
| Wolf & Muñoz (2014) | 1.Examine emotion recognition skills, including pain, of school-excluded boys | Cross-sectional  Correlational | Boys attending alternative short stay schools provided for youth who were permanently of temporarily expelled from their mainstream school because of continuous disruptive behavior | 100% | UK, Europe  (89.2% White British; 10.8% others) | CU | Two sets of dynamic stimuli presenting emotional facial expressions (4 videos for emotion, two male two female) and body postures (main body parts represented by patches of light) | Facial = 1s  Body = 3s | 1/24 (Facial)  1/20 (Body) | Identify the emotion | No | - |
| Woodworth & Waschbusch (2007) | 1.Examine the association between emotional processing, CP and CU traits in elementary school children | Cross-sectional  Between-groups | Participants were recruited via letters and trough fliers posted in public places | 80.82% | Canada, North America  (84.3% Caucasian; 4.3% African Canadian; 11.4% other ethnic categories) | CU | 18 pictures that included six photographs of a male face, six photographs of a female face and six drawing of a cartoon face. Each face depicted one emotion | NR | 1/18 | Identify the emotion | No | - |

*Note.* AA = African American; AB = Antisocial behavior; ACES = Assessment of Children’s Emotional Skills (Schultz et al., 2004); AO-CD = Adolescence-onset CD; ASD = Autism Spectrum Disorder; CD = Conduct disorder; CET = Child’s Eye Test (Baron-Cohen et al., 2001); CG = Comparison Group; CP = Conduct problems; CU = Callous-unemotional; CU+ = High levels of CU traits; CU- = Low levels of CU traits; DANVA-II = Diagnostic Analysis of Nonverbal Accuracy Scale 2 (Nowicki Jr & Carton, 1993); DBD = Disruptive behavior disorder; EA = European American; EI = Emotional intelligence; EMA = Ecological Momentary Assessment; EO-CD = Early-onset CD; ER = Emotion recognition; FELT = Facial Expression Labeling Task; FFSFP = Face-to-Face Still-Face Paradigm; FemNAT-CD (Kohls, Baumann et al. (2020); FERBT*:* Facial Expression Recognition Brazilian Task; GM = Grandiose-manipulative; HC = Healthy control; IAPS = International Affective Picture System; ICE = Increasingly Clear Emotions (Halberstadt et al., 2010); I/CP = Impulsivity/Conduct Problems; ICU = Inventory of Callous Unemotional Traits (Frick, 2004); INS = Impulsive-need of stimulation; LC = Low concern; LPE = Limited Prosocial Emotions; M *=* Media; MAPS = Multidimensional Assessment of Preschoolers; mCFS = Preattentive – processing paradigm modified (Adapted from Yang et al., 2007); MPAFC = Montreal Pain and Affective Face (Simon et al., 2006);  MSCEIT-YV-R = Mayer-Salovey-Caruso Emotional Intelligence Test-Youth Version-Research;  NimStim= NimStim Set of Facial Expressions (Tottenham et al., 2009); NC = Not clear; NR = Not reported; ODD = Oppositional defiant disorder; PDA = Personal Digital Assistant; PI = Punishment insensitivity; PP = Psychopathic traits/tendencies; PSD = Psychopathy Screening Device; RaFD = Radboud Faces Database (Langner et al., 2010); TI = Time Moment 1; T2 = Time Moment 2; T3 = Time Moment 3; T4 = Time Moment 4; The Emotion Hexagon task (Calder et al., 1996); The Expression Recognition Hexagon Stimuli (Calder et al., 1996); ToM = Theory of Mind; UK = United Kingdom; UNSW = University of New South Wales (Dadds et al., 2004); USA = United States of America; Vocal Affect Recognition Test (Scott et al., 1997).

**References contained on Table S3**

Baron-Cohen, S., Wheelwright, S., Hill, J., Raste, Y., & Plumb, I. (2001). The "Reading the mind in the eyes" Test revised version: A study with normal adults, and adults with Asperger syndrome or high-functioning autism. Journal of Child Psychology and Psychiatry, 42(2), 241–251. <https://doi.org/10.1111>

Calder, A. J., Young, A. W., Rowland, D., Perrett, D. I., Hodges, J. R., & Etcoff, N. L. (1996). Facial emotion recognition after bilateral amygdala damage: Differentially severe impairment of fear. *Cognitive Neuropsychology, 13*(5), 699–745. <https://doi.org/10.1080/026432996381890>

Dadds, M. R., Hawes, D. J. & Merz, S. (2004). *The UNSW Facial Emotion Task.* University of New South Wales.

Frick, P. J. (2004). *Inventory of Callous–Unemotional Traits.* APA PsycTests. <https://doi.org/10.1037/t62639-000>

Halberstadt, A. G., Leary, K. A., Garrett-Peters, P., Lozada, F. T., & Sibley, P. A. (2010). *The increasingly clear emotions (ICE) task*. Department of Psychology, North Carolina State University [Unpublished measure].

Kohls, G., Baumann, S., Gundlach, M., Scharke, W., Bernhard, A., Martinelli, A., Ackermann, K., Kersten, L., Prätzlich, M., Oldenhof, H., Jansen, L., van den Boogaard, L., Smaragdi, A., Gonzalez-Madruga, K., Cornwell, H., Rogers, J. C., Pauli, R., Clanton, R., Baker, R., . . . Konrad, K. (2020). Investigating sex differences in emotion recognition, learning, and regulation among youths with conduct disorder. *Journal of the American Academy of Child and Adolescent Psychiatry, 59*(2), 263-273. <https://doi.org/10.1016/j.jaac.2019.04.003>

Langner, O., Dotsch, R., Bijlstra, G., Wigboldus, D. H. J., Hawk, S. T., & van Knippenberg, A. (2010). Presentation and validation of the Radboud Faces Database. *Cognition and Emotion, 24*(8), 1377–1388. <https://doi.org/10.1080/02699930903485076>

Mayer, J. D., Salovey, P., & Caruso, D. R.  (2002). *Mayer–Salovey–Caruso emotional intelligence test (MSCEIT) user’s manual*. Multi-Health Systems.

Nowicki, S., Jr., & Carton, J. (1993). The measurement of emotional intensity from facial expressions. *The Journal of Social Psychology, 133*(5), 749 - 750. <https://doi.org/10.1080/00224545.1993.9713934>

Simon, D., Craig, K. D., Miltner, W. H., & Rainville, P. (2006). Brain responses to dynamic facial expressions of pain. *Pain, 126*(1-3), 309 – 318. https://doi.org/[10.1016/j.pain.2006.08.033](https://doi.org/10.1016/j.pain.2006.08.033)

Schultz, D., Izard, C. E., & Bear, G. G. (2004). Children's emotion processing: Relations to emotionality and aggression. *Development and Psychopathology, 16*(2), 371-387. <https://doi.org/10.1017/S0954579404044566>

Scott, S. K., Young, A. W., Calder, A. J., Hellawell, D.H.,Aggleton, J. P., & Johnson, M. (1997). *Impaired auditory recognition of fear and anger following bilateral amygdala lesions. Nature, 385*, 254-257. <https://doi.org/10.1038/385254a0>

Tottenham, N., Tanaka, J. W., Leon, A. C., McCarry, T., Nurse, M., Hare, T. A., Marcus, D. J., Westerlund, A., Casey, B. J., Nelson, C. (2009). The NimStim set of facial expressions: Judgments from untrained research participants. *Psychiatry Research, 168*(3), 242–249. <https://doi.org/10.1016/j.psychres.2008.05.006>

Yang, E., Zald, D. H., & Blake, R. (2007). Fearful expressions gain preferential access to awareness during continuous flash suppression. *Emotion, 7*, 882–886. <https://doi.org/10.1037/1528-3542.7.4.882>

Tabla S4

*Studies´s Quality Assessment by Using the Appraisal Tool for Cross-sectional Studies (AXIS)*

| **Reference** | **1** | **2** | **3** | **4** | **5** | **6** | **7** | **8** | **9** | **10** | **11** | **12** | **13^a^** | **14** | **15** | **16** | **17** | **18** | **19^a^** | **20** | **Total score** |
| --- | --- | --- | --- | --- | --- | --- | --- | --- | --- | --- | --- | --- | --- | --- | --- | --- | --- | --- | --- | --- | --- |
| Aghanjani et al. (2021) | 1 | 1 | 0 | 1 | 1 | 1 | 0 | 1 | 1 | 1 | 1 | 1 | 0 | 0 | 1 | 1 | 1 | 1 | 1 | 1 | 16 |
| Bennet & Kerig (2014) | 1 | 1 | 0 | 1 | 1 | 1 | 0 | 1 | 1 | 1 | 1 | 1 | 1 | 0 | 1 | 1 | 1 | 1 | 0 | 1 | 16 |
| Billeci et al. (2018) | 1 | 1 | 0 | 1 | 1 | 1 | 0 | 1 | 1 | 1 | 1 | 1 | 1 | 0 | 1 | 1 | 1 | 1 | 1 | 1 | 17 |
| Blair y Coles (2000) | 1 | 1 | 0 | 1 | 1 | 1 | 0 | 1 | 1 | 1 | 1 | 1 | 1 | 0 | 1 | 1 | 1 | 1 | 0 | 1 | 16 |
| Blair et al. (2001) | 1 | 1 | 0 | 1 | 1 | 1 | 0 | 1 | 1 | 1 | 1 | 1 | 1 | 0 | 1 | 1 | 1 | 1 | 0 | 1 | 16 |
| Blair et al. (2005) | 1 | 1 | 0 | 1 | 1 | 1 | 0 | 1 | 1 | 1 | 1 | 1 | 1 | 0 | 1 | 1 | 1 | 1 | 0 | 1 | 16 |
| Bours et al. (2018) | 1 | 1 | 0 | 1 | 1 | 1 | 0 | 1 | 1 | 1 | 1 | 1 | 1 | 0 | 1 | 1 | 1 | 1 | 0 | 1 | 17 |
| Bowen et al. (2014) | 1 | 1 | 0 | 1 | 1 | 1 | 0 | 1 | 1 | 1 | 1 | 1 | 1 | 1 | 1 | 1 | 1 | 1 | 0 | 1 | 17 |
| Dadds et al. (2006) | 1 | 1 | 1 | 1 | 1 | 1 | 0 | 1 | 1 | 1 | 1 | 1 | 1 | 0 | 1 | 1 | 1 | 0 | 1 | 1 | 17 |
| Dadds et al. (2008) | 1 | 1 | 0 | 1 | 1 | 1 | 0 | 1 | 1 | 1 | 1 | 1 | 1 | 0 | 1 | 1 | 1 | 0 | 1 | 1 | 15 |
| Dadds et al. (2011) | 1 | 1 | 0 | 1 | 1 | 1 | 0 | 1 | 1 | 1 | 1 | 1 | 1 | 0 | 1 | 1 | 1 | 1 | 0 | 1 | 16 |
| Dadds et al. (2018) | 1 | 1 | 0 | 1 | 1 | 1 | 0 | 1 | 1 | 1 | 1 | 0 | 1 | 0 | 1 | 1 | 1 | 1 | 0 | 1 | 15 |
| Demetriu &Fanti (2022) | 1 | 1 | 0 | 1 | 1 | 1 | 0 | 1 | 1 | 1 | 1 | 1 | 0 | 1 | 1 | 1 | 1 | 1 | 0 | 1 | 16 |
| Ezpleta et al. (2017) | 1 | 1 | 0 | 1 | 1 | 1 | 0 | 1 | 0 | 1 | 1 | 0 | 1 | 0 | 1 | 1 | 1 | 0 | 1 | 1 | 14 |
| Fairchild et al. (2009) | 1 | 1 | 0 | 1 | 1 | 1 | 0 | 1 | 1 | 1 | 1 | 1 | 1 | 0 | 1 | 1 | 1 | 1 | 1 | 1 | 17 |
| Fairchild et al. (2010) | 1 | 1 | 0 | 1 | 1 | 1 | 0 | 1 | 1 | 1 | 1 | 1 | 1 | 0 | 1 | 1 | 1 | 0 | 1 | 1 | 16 |
| Gillen et al. (2018) | 1 | 1 | 0 | 1 | 1 | 1 | 0 | 1 | 1 | 1 | 1 | 1 | 1 | 0 | 1 | 1 | 1 | 1 | 1 | 1 | 18 |
| Hartmann & Schwenck (2020) | 1 | 1 | 0 | 1 | 1 | 1 | 1 | 1 | 1 | 1 | 1 | 1 | 1 | 1 | 1 | 1 | 1 | 1 | 1 | 1 | 19 |

| **Reference** | **1** | **2** | **3** | **4** | **5** | **6** | **7** | **8** | **9** | **10** | **11** | **12** | **13^a^** | **14** | **15** | **16** | **17** | **18** | **19**^a^ | **20** | **Total score** |
| --- | --- | --- | --- | --- | --- | --- | --- | --- | --- | --- | --- | --- | --- | --- | --- | --- | --- | --- | --- | --- | --- |
| Kahn et al. (2016) | 1 | 1 | 0 | 1 | 1 | 1 | 0 | 1 | 1 | 1 | 1 | 1 | 1 | 0 | 1 | 1 | 1 | 1 | 1 | 1 | 17 |
| Kahn et al. (2017) | 1 | 1 | 1 | 1 | 1 | 1 | 0 | 1 | 0 | 1 | 1 | 1 | 0 | 0 | 0 | 1 | 0 | 1 | 1 | 1 | 16 |
| Kimonis et al. (2016) | 1 | 1 | 1 | 1 | 1 | 1 | 1 | 1 | 1 | 1 | 1 | 1 | 1 | 1 | 1 | 1 | 1 | 1 | 1 | 1 | 20 |
| Klapwijk et al. (2016) | 1 | 1 | 0 | 1 | 1 | 1 | 0 | 1 | 1 | 1 | 1 | 1 | 1 | 0 | 1 | 1 | 1 | 1 | 1 | 1 | 17 |
| Kohls, Baumann et al. (2020) | 1 | 1 | 0 | 1 | 1 | 1 | 0 | 1 | 1 | 1 | 1 | 1 | 1 | 0 | 1 | 1 | 1 | 1 | 1 | 1 | 17 |
| Kohls, Fairchild et al. (2020) | 1 | 1 | 0 | 1 | 1 | 1 | 0 | 1 | 1 | 1 | 1 | 1 | 1 | 0 | 1 | 1 | 1 | 1 | 1 | 1 | 17 |
| Lemos -Vasconcellos et al. (2014) | 1 | 1 | 0 | 0 | 1 | 1 | 0 | 1 | 1 | 0 | 0 | 0 | 1 | 0 | 1 | 1 | 1 | 1 | 1 | 0 | 12 |
| Levantini et al. (2022) | 1 | 1 | 1 | 1 | 1 | 1 | 0 | 1 | 1 | 1 | 1 | 1 | 1 | 0 | 1 | 1 | 1 | 1 | 1 | 1 | 18 |
| Lui et al. (2016) | 1 | 1 | 0 | 1 | 1 | 1 | 0 | 1 | 1 | 1 | 1 | 1 | 1 | 0 | 1 | 1 | 1 | 1 | 1 | 1 | 17 |
| Martin – Key y Fairchild (2017) | 1 | 1 | 0 | 1 | 1 | 1 | 0 | 1 | 1 | 1 | 1 | 1 | 1 | 0 | 1 | 1 | 1 | 1 | 1 | 1 | 17 |
| Martin-Key et al. (2018) | 1 | 1 | 0 | 1 | 1 | 1 | 1 | 1 | 1 | 1 | 1 | 1 | 1 | 1 | 1 | 1 | 1 | 1 | 1 | 1 | 19 |
| Martin-Key et al. (2020) | 1 | 1 | 0 | 1 | 1 | 1 | 1 | 1 | 1 | 1 | 1 | 1 | 1 | 1 | 1 | 1 | 1 | 1 | 1 | 1 | 19 |
| Martin – Key et al. (2021) | 1 | 1 | 0 | 1 | 1 | 1 | 0 | 1 | 1 | 1 | 1 | 1 | 1 | 0 | 1 | 1 | 1 | 1 | 1 | 1 | 18 |
| Milone et al. (2019) | 1 | 1 | 0 | 1 | 1 | 1 | 0 | 1 | 1 | 1 | 0 | 0 | 1 | 0 | 0 | 0 | 0 | 1 | 1 | 1 | 13 |
| Moore et al. (2019) | 1 | 1 | 0 | 1 | 1 | 1 | 0 | 1 | 1 | 1 | 0 | 1 | 1 | 0 | 0 | 1 | 1 | 1 | 1 | 1 | 15 |
| Muñoz. (2009) | 1 | 1 | 0 | 1 | 1 | 1 | 0 | 1 | 1 | 1 | 0 | 1 | 1 | 0 | 1 | 1 | 1 | 0 | 1 | 1 | 15 |
| Muñoz et al. (2021) | 1 | 1 | 0 | 1 | 1 | 1 | 1 | 1 | 1 | 1 | 1 | 1 | 1 | 1 | 1 | 1 | 1 | 1 | 1 | 1 | 19 |
| O’Kearney et al. (2017) | 1 | 1 | 0 | 1 | 1 | 1 | 0 | 1 | 1 | 1 | 1 | 0 | 1 | 0 | 1 | 1 | 1 | 1 | 1 | 1 | 16 |
| O`Kearney et al. (2020) | 1 | 1 | 0 | 1 | 1 | 1 | 0 | 1 | 1 | 1 | 1 | 0 | 1 | 0 | 1 | 1 | 1 | 0 | 1 | 1 | 15 |
| Pauli et al. (2021) | 1 | 1 | 0 | 1 | 1 | 1 | 1 | 1 | 1 | 1 | 1 | 1 | 1 | 0 | 1 | 1 | 1 | 1 | 1 | 1 | 18 |

| **Reference** | **1** | **2** | **3** | **4** | **5** | **6** | **7** | **8** | **9** | **10** | **11** | **12** | **13^a^** | **14** | **15** | **16** | **17** | **18** | **19**^a^ | **20** | **Total score** |
| --- | --- | --- | --- | --- | --- | --- | --- | --- | --- | --- | --- | --- | --- | --- | --- | --- | --- | --- | --- | --- | --- |
| Schwenck et al. (2011) | 1 | 1 | 0 | 1 | 1 | 1 | 0 | 1 | 0 | 1 | 1 | 1 | 0 | 0 | 1 | 1 | 1 | 1 | 1 | 1 | 15 |
| Schwenk et al. (2014) | 1 | 1 | 0 | 1 | 1 | 1 | 0 | 1 | 1 | 1 | 1 | 1 | 0 | 0 | 1 | 1 | 1 | 1 | 1 | 1 | 15 |
| Sharp et al. (2014) | 1 | 1 | 0 | 0 | 1 | 1 | 1 | 1 | 1 | 0 | 1 | 1 | 1 | 0 | 1 | 1 | 1 | 1 | 1 | 1 | 16 |
| Stevens et al. (2001) | 1 | 1 | 0 | 1 | 1 | 1 | 0 | 1 | 1 | 0 | 1 | 1 | 0 | 0 | 1 | 1 | 1 | 0 | 1 | 1 | 14 |
| Sylvers et al. (2011) | 1 | 1 | 0 | 1 | 1 | 1 | 0 | 1 | 1 | 1 | 1 | 0 | 0 | 0 | 1 | 1 | 1 | 1 | 1 | 1 | 15 |
| White et al. (2016) | 1 | 1 | 1 | 1 | 1 | 1 | 1 | 1 | 1 | 1 | 1 | 1 | 1 | 0 | 1 | 1 | 1 | 0 | 1 | 1 | 18 |
| Wolf & Muñoz (2014) | 1 | 1 | 0 | 1 | 1 | 1 | 0 | 1 | 1 | 0 | 1 | 1 | 1 | 0 | 1 | 1 | 1 | 1 | 1 | 1 | 16 |
| Woodworth y Waschbusch (2007) | 1 | 1 | 0 | 1 | 1 | 1 | 0 | 1 | 1 | 1 | 1 | 1 | 0 | 1 | 1 | 1 | 1 | 1 | 1 | 1 | 18 |

*Note.* Items: 1.Aims; 2.Study Design; 3.Sample Size Justification; 4.Target population; 5.Sampling frame; 6.Sample selection; 7.Non-responders; 8.Measurement of risk and outcome variables; 9.Measurement validity and reliability; 10.Statistics; 11.Method replication; 12.Basic data; 13.Response rate; 14.Information about non-responders; 15.Internally consistent results; 16.Analysis results; 17.Justified discussions and conclusions; 18.Limitations; 19.Funding sources/ conflicts of interest; 20.Ethical approval. Coding: 1= YES; 0= NO/no sufficient data

^a^ Reversed scores (1= NO/ no sufficient data; 0= YES)

Tabla S5

*Studies´s Quality Assessment by Using the Critical Appraisal Skills Program (CASP) for Longitudinal Studies*

| **Reference** | **1** | **2** | **3** | **4** | **5a** | **5b** | **6** | **6b** | **7** | **8** | **9** | **10** | **11** | **12** |
| --- | --- | --- | --- | --- | --- | --- | --- | --- | --- | --- | --- | --- | --- | --- |
| Bedford et al. (2017) | YES | YES | YES | YES | YES | YES | CT | YES | YES | YES | YES | YES | YES | YES |
| De Ridder et al. (2016) | YES | YES | NO | NO | NO | YES | CT | NO | YES | YES | YES | NO | YES | YES |
| Peticlerc et al. (2019) | YES | YES | YES | YES | YES | YES | YES | YES | YES | YES | YES | YES | YES | YES |
| Redher et al. (2017) | YES | YES | YES | YES | NO | YES | CT | NO | YES | YES | YES | NO | YES | YES |
| Schuberth et al. (2018) | YES | YES | YES | YES | NO | YES | CT | YES | YES | YES | YES | NO | YES | YES |

*Note.* Items: 1.Address clearly focused issue; 2.Cohort recruited in an acceptable way; 3.Exposure accurately measured to minimise bias; 4.Outcome accurately measured to minimise bias; 5a.Identified confounding factors; 5b. Consider the confounding factors in the design; 6a.Follow up of subjects; 6b.Follow up of subject long enough; 7.Results of the study; 8.Precision of results; 9.Credibility of results; 10.Results can be applied to local population; 11.The study fit with other available evidence; 12. Implications of this study for practise

**Table S6**

*Excluded Items after Abstract and Full Text Review and Reasons (k=24)*

| Criterion | | Reference |
| --- | --- | --- |
| 1. Age | Exceeded the age criterion (mean age > 18) (*k*=3) | Halty, L. (2019). Impairment in the processing of fear gaze in adolescents with callous-unemotional traits. *Psychology, Crime &amp; Law, 25*(8), 792-802. <https://doi.org/10.1080/1068316X.2019.1588970> |
|  |  | Kyranides, M. N., Petridou, M., Gokani, H. A., Hill, S., & Fanti, K. A.Reading and reacting to faces, the effect of facial mimicry in improving facial emotion recognition in individuals with antisocial behavior and psychopathic traits. *Current Psychology,* <https://doi.org/10.1007/s12144-022-02749-0> |
|  |  | Lethbridge, E. M., Richardson, P., Reidy, L., y Taroyan, N. A. (2017). Exploring the relationship between callous-unemotional traits, empathy processing and affective valence in a general population. *Journal of Psychology, 13*(1), 162-172. <https://doi.org/10.5964/ejop.v13i1.1179> |
| 1. Objectives and method | Measured emotion recognition through questionnaires or scenarios with contextual cues (*k*=3) | Ezpeleta, L., Granero, R., de la Osa, N., y Domènech, J. M. (2017). Developmental trajectories of callous-unemotional traits, anxiety and oppositionality in 3–7-year-old children in the general population. *Personality and Individual Differences, 111*, 124-133. <https://doi.org/><http://dx.doi.org/10.1016/j.paid.2017.02.005> |
|  |  | Herpers, P. C. M., Bakker-Huvenaars, M. J., Greven, C. U., Wiegers, E. C., Nijhof, K. S., Baanders, A. N., Buitelaar, J. K., & Rommelse, N. N. J. (2019). Emotional valence detection in adolescents with oppositional defiant disorder/conduct disorder or autism spectrum disorder. *European Child & Adolescent Psychiatry, 28*(7), 1011-1022. <https://doi.org/10.1007/s00787-019-01282-z> |
|  |  | White, B. A., Dede, B., Heilman, M., Revilla, R., Lochman, J., Hudac, C. M., Bui, C., & White, S. W. (2022). Facial affect sensitivity training for young children with emerging CU traits: An experimental therapeutics approach. *Journal of Clinical Child and Adolescent Psychology : The Official Journal for the Society of Clinical Child and Adolescent Psychology, American Psychological Association, Division 53, 51*(3), 264-276. <https://doi.org/10.1080/15374416.2022.2056895> |

| Criterion | | Reference |
| --- | --- | --- |
|  | Included complex instead of basic emotions (*k* = 1) | Morosan, L., Badoud, D., Zaharia, A., Brosch, T., Eliez, S., Bateman, A., Heller, P., & Debbané, M. (2017). *Emotion recognition and perspective taking: A comparison between typical and incarcerated male adolescents*. Public Library of Science (PLoS). <https://doi.org/10.1371/journal.pone.0170646> |
|  | Emotion recognition computed as a function of a global measure (*k* = 5) | De la Osa, N., Granero, R., Domenech, J. M., Shamay-Tsoory, S., & Ezpeleta, L. (2016). Cognitive and affective components of theory of mind in preschoolers with oppositional defiance disorder: Clinical evidence. *Psychiatry Research, 241*, 128-134. <https://doi.org/10.1016/j.psychres.2016.04.082> |
|  |  | Fragkaki, I., Cima, M. J., y Meesters, C. (2016). The association between callous-unemotional traits, externalizing problems, and gender in predicting cognitive and affective morality judgments in adolescence. *Journal of Youth and Adolescence, 45*(9), 1917-1930. <https://doi.org/10.1007/s10964-016-0527-x> |
|  |  | Jones, A. P., Larsson, H., Ronald, A., Rijsdijk, F., Busfield, P., Mcmillan, A., Plomin, R., & Viding, E. (2009). Phenotypic and aetiological associations between psychopathic tendencies, autistic traits, and emotion attribution. *Criminal Justice and Behavior, 36*(11), 1198-1212. <https://doi.org/10.1177/0093854809342949> |
|  |  | Northam, J. C., Kurukulasuriya, N., Hunt, C., & Hawes, D. J. (2022). Moral reasoning, emotion understanding, and callous-unemotional traits in early-to-middle childhood. *The British Journal of Developmental Psychology, 40*(2), 306-319. <https://doi.org/10.1111/bjdp.12402> |
|  |  | Schuberth, D. A., Zheng, Y., Pasalich, D. S., McMahon, R. J., Kamboukos, D., Dawson-McClure, S., & Brotman, L. M. (2019). The role of emotion understanding in the development of aggression and callous-unemotional features across early childhood. *Journal of Abnormal Child Psychology, 47*(4), 619-631. <https://doi.org/10.1007/s10802-018-0468-9> |
|  | Emotion recognition restricted to emotional responsiveness (*k* = 2) | Baroncelli, A., Roti, B., y Ciucci, E. (2018). The associations between callous-unemotional traits and emotional awareness in youth. *Personality and Individual Differences, 120*, 247-252. <https://doi.org/10.1016/j.paid.2017.07.036> |
|  |  | Northam, J. C., Dar, H., Hawes, D. J., Barnes, K., McNair, N. A., Fisher, C. A., & Dadds, M. R. More than a feeling? an expanded investigation of emotional responsiveness in young children with conduct problems and callous-unemotional traits. *Development and Psychopathology,* <https://doi.org/10.1017/S0954579421001590> |

| Criterion | | Reference |
| --- | --- | --- |
|  | Emotion recognition not assessed in relation to psychopathic traits (*k* = 4) | Aghajani, M., Klapwijk, E. T., Colins, O. F., Ziegler, C., Domschke, K., Vermeiren, R. R., y van der Wee, N. J. (2018). Interactions between oxytocin receptor gene methylation and callous-unemotional traits impact socioaffective brain systems in conduct-disordered offenders. *Biological Psychiatry. Cognitive Neuroscience and Neuroimaging, 3*(4), 379-391. <https://doi.org/S2451-9022(18)30002-8> |
|  |  | Drabick, D., Jakubovic, R. J., Everett, V. S., Friedman, A. L., Emory, G. O., & Kalchthaler, F. B. (2021). Conduct problems among children in low-income, urban neighborhoods: A developmental psychopathology- and RDoC-informed approach. *Development and Psychopathology, 33*(5), 1864-1881. <https://doi.org/10.1017/S0954579421001103> |
|  |  | Thijssen, J., Otgaar, H., Meijer, E. H., Smeets, T., y de Ruiter, C. (2012). Emotional memory for central and peripheral details in children with callous-unemotional traits. *Behavioral Sciences the Law, 30*(4), 506-515. <https://doi.org/10.1002/bsl.2021> |
|  |  | Von Polier, G.G. Greimel, E., Konrad, K., GroBheinrich, N., Kohls, G., Vloet, T.D…. y Schulte-Rüther, M. (2020). Neural correlates of empathy in boys with early onset conduct problems. *Frontiers in Psychiatry, 11:178.* <https://doi.org/10.3389/fpsyt.2020.00178> |
|  | Measured emotional processing instead of emotion recognition (*k* = 4) | de Wied, M., Meeus, W., & van Boxtel, A.Disruptive behavior disorders and psychopathic traits in adolescents: Empathy-related responses to witnessing animal distress. *Journal of Psychopathology and Behavioral Assessment,* <https://doi.org/10.1007/s10862-021-09891-2> |
|  |  | Kimonis, E. R., Frick, P. J., Fazekas, H., & Loney, B. R. (2006). Psychopathy, aggression, and the processing of emotional stimuli in non-referred girls and boys. *Behavioral Sciences &amp; the Law, 24*(1), 21-37. <https://doi.org/10.1002/bsl.668> |
|  |  | Levantini, V., Muratori, P., Calderoni, S., Inguaggiato, E., Masi, G., Milone, A., Tonacci, A., & Billeci, L. (2022). Parenting practices moderate the link between attention to the eyes and callous unemotional traits in children with disruptive behavior disorder: An eye-tracking study. *Journal of Psychiatric Research, 146*, 272-278. <https://doi.org/S0022-3956(21)00665-8> |
|  |  | Masi, G., Milone, A., Pisano, S., Lenzi, F., Muratori, P., Gemo, I., Bianchi, L., Mazzone, L., Postorino, V., Sanges, V., Williams, R., & Vicari, S. (2014). Emotional reactivity in referred youth with disruptive behavior disorders: The role of the callous-unemotional traits. *Psychiatry Research, 220*(1), 426-432. <https://doi.org/10.1016/j.psychres.2014.07.035> |

| Criterion | | Reference |
| --- | --- | --- |
| 1. Co-founding variables (k = 2) | Evidence of substance abuse (k = 2) | Blair, R., Bashford-Largo, J., Zhang, R., Mathur, A., Schwartz, A., Elowsky, J., Tyler, P., Hammond, C. J., Filbey, F. M., Dobbertin, M., Bajaj, S., & Blair, K. S. (2021). Alcohol and cannabis use disorder symptom severity, conduct disorder, and callous-unemotional traits and impairment in expression recognition. *Frontiers in Psychiatry, 12* <https://doi.org/10.3389/fpsyt.2021.714189> |
|  |  | Leist, T., y Dadds, M. R. (2009). Adolescent´s ability to read different emotional faces relates to their history of maltreatment and type of psychopathology. *Clinical Child Psychology and Psychiatry, 14*(2), 237-250. <https://doi.org/10.1177/1359104508100887> |
